# Effects of Afforestation Restoration on Soil Potential N_2_O Emission and Denitrifying Bacteria After Farmland Abandonment in the Chinese Loess Plateau

**DOI:** 10.3389/fmicb.2019.00262

**Published:** 2019-02-19

**Authors:** Na Deng, Honglei Wang, Shu Hu, Juying Jiao

**Affiliations:** State Key Laboratory of Soil Erosion and Dryland Farming on the Loess Plateau, Institute of Soil and Water Conservation, Northwest A&F University, Yangling, China

**Keywords:** denitrification, nitrous oxide, high-throughput sequencing, afforestation restoration, denitrifying bacteria, farmland abandonment, Loess Plateau

## Abstract

Denitrification is a critical component of soil nitrogen (N) cycling, including its role in the production and loss of nitrous oxide (N_2_O) from the soil system. However, restoration effects on the contribution of denitrification to soil N_2_O emissions, the abundance and diversity of denitrifying bacteria, and relationships among N_2_O emissions, soil properties, and denitrifying bacterial community composition remains poorly known. This is particularly true for fragile semiarid ecosystems. In order to address this knowledge gap, we utilized 42-year chronosequence of *Robinia pseudoacacia* plantations in the Chinese hilly gullied Loess Plateau. Soil potential N_2_O emission rates were measured using anaerobic incubation experiments. Quantitative polymerase chain reaction (Q-PCR) and Illumina MiSeq high-throughput sequencing were used to reveal the abundance and community composition of denitrifying bacteria. In this study, the afforestation practices following farmland abandonment had a strong negative effect on soil potential N_2_O emission rates during the first 33 years. However, potential N_2_O emission rates steadily increased in 42 years of restoration, leading to enhanced potential risk of greenhouse gas emissions. Furthermore, active afforestation increased the abundance of denitrifying functional genes, and enhanced microbial biomass. *Actinobacteria* and *Proteobacteria* were the dominant denitrifying bacterial phyla in the 0 to 33-years old sites, while the 42-years sites were dominated by *Planctomycetes* and *Actinobacteria*, implying that the restoration performed at these sites promoted soil microbial succession. Finally, correlation analyses revealed that soil organic carbon concentrations had the strongest relationship with potential N_2_O emission rates, followed by the abundance of the *nosZ* functional gene, bulk density, and the abundance of *Bradyrhizobium* and *Variovorax* across restoration stages. Taken together, our data suggest above-ground restoration of plant communities results in microbial community succession, improved soil quality, and significantly altered N_2_O emissions.

## Introduction

Nitrogen management is one of the major environmental challenges for the 21st century (Sutton et al., 2011), and the mitigation of nitrous oxide (N_2_O) emissions are of particular concern due to the strong role N_2_O can play in climate change. In fact, the contribution of N_2_O as a greenhouse gas affecting global temperatures is third only to CO_2_ and CH_4_, which have global warming potentials approximately 298 and 11.9 times larger than N_2_O, respectively (Domeignoz-Horta et al., 2018). N_2_O is also involved in the destruction of the stratospheric ozone layer (Crutzen, 1970) and has become the dominant ozone depleting substance (Ravishankara et al., 2009). With N_2_O emissions on the rise [12–16 Tg N yr-1 from 2000 to 2050 (Bouwman et al., 2013; Hu et al., 2015)], studies that explore the factors that regulate N_2_O fluxes for soil, ocean, estuaries, freshwater habitats, and wastewater treatment plants are increasing (Schreiber et al., 2012; Zhang Y. et al., 2016).

Soils constitute the largest source of N_2_O emissions (Bremner and Blackmer, 1979; Hu et al., 2015), especially in agricultural and forested ecosystems (Kong et al., 2010; Smethurst, 2010; Levy-Booth et al., 2014). To enable more effective mitigation to counteract the steady increase in N_2_O emissions, it is necessary to better understand the mechanisms that drive N_2_O fluxes in different ecosystems (Hu et al., 2015). Numerous studies have focused on estimation and simulation of N_2_O fluxes (Reay et al., 2012), assessing the impact of environmental factors on N_2_O efflux (e.g., Bateman and Baggs, 2005; Chen et al., 2012; Németh et al., 2014), the central role of soil microbial communities in regulating nitrogen cycle processes and N_2_O emissions (e.g., Baggs, 2011; Bouwman et al., 2013; Tatti et al., 2013; Chen et al., 2015) and microbial ecology that helps determine the production and consumption of N_2_O (Barberan et al., 2012; Singh et al., 2010). Specifically, denitrification is one of the major microbial pathways fueling N_2_O emissions from soil (Smith et al., 2003; Shaw et al., 2006; Levy-Booth et al., 2014). Denitrification consists of sequential reductions of soluble NO_3_^-^ and NO_2_^-^ to the nitrogen gases NO, N_2_O, and N_2_ via four enzymatic complexes (Philippot et al., 2011). For instance, the first step (NO_3_^-^ → NO_2_^-^) is catalyzed by nitrate reductase encoded by *napA* and *narG* genes; the second step (NO_2_^-^ → NO) is catalyzed by *nirK* and *nirS* genes encoding nitrite reductase; the third step leading to N_2_O production (NO → N_2_O) is mediated by nitrous reductase, encoded by *qnorB* gene. And the last step, the reduction of N_2_O to N_2_ occurs through the *nosZ* gene encoding nitrous reductase, which is the only known sink for N_2_O in the biosphere (Philippot et al., 2007). Denitrification is an enzymatically catalyzed process and can be strongly influenced by environmental factors, including soil conditions (Smith et al., 2003; Cuhel et al., 2010; Hu et al., 2014; Levy-Booth et al., 2014) and vegetation features (Zhang C. et al., 2016; Liu et al., 2018). For example, several studies have reported increasing N_2_O emissions with decreasing soil pH (Bergaust et al., 2010; Cuhel et al., 2010) and oxygen concentrations (Chapuis-Lardy et al., 2007; Schreiber et al., 2012). Those effects have been attributed to a specific regulation of N_2_O reductase activity (Richardson et al., 2009). Changes in soil pH and oxygen concentration can be influenced by several factors depending on vegetation type, land-use history, and time since active restoration occurred (Cheng and An, 2015; Kou et al., 2016; Nadal-Romero et al., 2016). This supports the hypothesis that vegetation, edaphic properties, and microbial community are closely linked over the course of ecosystem succession following disturbance (Lozano et al., 2014; Zhang C. et al., 2016; Barber et al., 2017). However, the rules that govern these interacting associations remain relatively poorly understood (Bernhard and Kelly, 2016), and few studies focus on the correlation between N_2_O emission rates and the abundances, composition, and diversity of denitrifying bacterial community during vegetation management (e.g., afforestation) of abandoned farmland in semiarid regions over the long-term.

Due to the high degree of human activities that have occurred over the long-term, such as cultivation and grazing, the Chinese Loess Plateau had been one of the most eroded regions and one of the most vulnerable areas to desertification in China (Fu et al., 2011). To address this issue, a number of programs were initiated by the Chinese government in the 20st century, for example, “Grain for Green,” which was the conversion of steep cultivated land to forest and grassland. In particular, afforestation was considered as a key restoration approach in China for the past few decades (Kou et al., 2016; Hu et al., 2017) and, due to its tolerance of a wide range of soil conditions (Liu et al., 2018), the nitrogen-fixing tree *Robinia pseudoacacia* has been widely planted as a pioneer afforestation species in the Chinese Loess Plateau. To date, the effects of this restoration on a vegetation competition, soil properties, and microbial communities during afforestation have been reported (Jiao et al., 2012; Cheng and An, 2015; Kou et al., 2016; Zhang C. et al., 2016; Liu et al., 2018). However, studies of N_2_O emissions and the denitrifying microbial communities that drive these emissions during afforestation are still few, especially in arid and semiarid ecosystems.

In order to address this important knowledge gap, here we simultaneously assessed multiple aspects of plant and soil in a restoration chronosequence in the Chinese Loess Plateau. The goals of our study were to (1) assess the potential rates of N_2_O efflux across reforested sites of different ages, (2) determine the abundance of denitrifying functional genes and discern the diversity and composition of total bacteria and denitrifying bacteria communities, and (3) explore the relationships among potential N_2_O emission rates, soil properties, and denitrifying bacterial community across a range of restoration stages.

## Materials and Methods

### Study Area

This study was conducted in the Zhifanggou watershed (36°43′21″–36°46′10″N, 109°14′26″–109°15′44″E), located in the hilly gullied region of the Chinese Loess Plateau. Climate is semiarid, characterized by a mean annual temperature of 8.8°C (with a mean minimum temperature in January of -6.2°C and a mean maximum temperature in August of 37.2°C). Mean annual precipitation is 504 mm and a mean annual evaporation is 1,000 mm (Kou et al., 2016; Zhang C. et al., 2016). The soils are mainly Calcaric Cambisols, and the study area is distributed in the transition zone between forest and steppe. Species located in the area include *Sophora viciifolia, Periploca sepium, Rosa xanthina, Spiraea pubescens, Artemisia scoparia, Lespedeza davurica, Stipa bungeana, Artemisia giraldii, Artemisia gmelinii*, and *Bothriochloa ischaemun*.

Historical farming practices at the sites removed native species, resulting in severe soil erosion and land degradation (Fu et al., 2011). The primary approach to solve these problems has been vegetation restoration (conversion of steep cultivated land to forest and grassland) (Zheng, 2006). In this context, *R. pseudoacacia* was considered as a pioneer tree for afforestation and more than 70,000 ha of land was reforested with *R. pseudoacacia* from 1950 to 2005 in the Chinese Loess Plateau (Jiao et al., 2012; Kou et al., 2016). To investigate the restoration effects of *R. pseudoacacia* plantations on N_2_O emission and denitrifier communities, the method of space for time substitution was used in our study. We selected *R. pseudoacacia* plantations of different ages (14, 26, 33, and 42-years), and farmland (0-year) for the control. All the *R. pseudoacacia* plantations had been planted with a layout of plantings that were 2 m × 2 m, resulting in similar initial tree densities. The ages of the *R. pseudoacacia* plantations were obtained from interviews with local farmers and compared with records registered with the An’sai Ecological Experimental Station of Soil and Water Conservation. Farmland agricultural practices included planting potatoes (*Solanum tuberosum*) and fertilizing with 600–900 kg ha^-1^ N urea and 400–600 kg ha^-1^ phosphorus pentoxide (P_2_O_5_) per year. *R. pseudoacacia* plantations and farmland control sites were selected with three replicates per restoration stage. Each of the three replicates was on a different hill slope. All sites were south-facing, and soil properties of the studied sites are presented in Table 1 and Supplementary Table S1.

**Table 1:** Soil physicochemical properties across different restoration n stages.

Sites	Bulk density (g cm^-3^)	pH	Organic C (g kg^-1^)	Total N (g kg^-1^)	Ammonium (mg kg^-1^)	Nitrate (mg kg^-1^)
0-year	1.22 ± 0.02a	8.28 ± 0.05c	3.84 ± 0.05e	0.44 ± 0.01e	35.04 ± 0.67a	12.77 ± 0.03a
14-year	1.15 ± 0.01b	8.38 ± 0.05b	5.72 ± 0.07d	0.61 ± 0.01c	4.24 ± 0.11c	13.01 ± 0.39a
26-year	1.13 ± 0.01b	8.30 ± 0.02c	12.93 ± 0.46b	1.04 ± 0.01a	4.56 ± 0.52c	14.96 ± 1.49a
33-year	1.06 ± 0.02c	8.54 ± 0.01a	15.17 ± 0.10a	0.47 ± 0.01d	4.82 ± 0.41c	5.31 ± 0.17c
42-year	0.99 ± 0.03d	8.39 ± 0.01b	10.59 ± 0.02c	0.72 ± 0.02b	6.67 ± 0.30b	6.90 ± 0.07b


*Different letters within a row indicate significant differences (*P* < 0.05) among restoration stages based on a one-way ANOVA, followed by an LSD test.*

### Vegetation Survey

A vegetation survey was conducted on our study sites in July 2016 (the peak of the growing season in the area). In each site, a 10 m × 10 m quadrant was established to estimate the canopy density and the vegetation cover, and five 1 m × 1 m quadrants were randomly selected to identify all plant species and record the number of individuals of each plant species. The canopy density and vegetation cover were estimated visually by three observers. The numbers of plant species were used to calculate the Pielou evenness index and Shannon diversity index, which were used to estimate the evenness and diversity, respectively (Kou et al., 2016). The number of species was used to estimate the richness (Supplementary Tables S2, S3).

### Soil Sampling

Soil samples were collected in July 2016, when the soil moisture and temperature are conducive to microorganism activity (Bateman and Baggs, 2005). In each site, we sampled to a depth of 20 cm because most of the soil nutrients and microorganisms are in this upper soil layer (Kou et al., 2016). We then took three samples in each site, with each sample made up of six evenly distributed cores (5 cm in diameter and 20 cm deep). These soils were used to determine the organic carbon content, total nitrogen content, extractable ammonium and nitrate content, pH, potential N_2_O emission rates, and the abundance and composition of soil denitrifying bacteria. The litter horizons were removed before soil sampling was performed. Soil samples were kept sealed and in a cooler with ice prior to analysis. After transportation to the laboratory, the soil samples were immediately sieved (< 2 mm), and visible plant roots, stones, and debris were removed. Soils were then separated into two subsamples: one subsample was immediately stored at -80°C for DNA analysis, and the other sample was air-dried at room temperature for chemo-physical analyses. Furthermore, another three evenly distributed cores (height = 5 cm, diameter = 5.05 cm) were sampled to determine soil bulk density in each site.

### Soil Analysis

#### N_2_O Emission Experiment

Laboratory incubations were used to determine potential N_2_O emissions rates. The pathways of N_2_O emission are strongly influenced by soil temperature and moisture (Bateman and Baggs, 2005). In our study area, the *in situ* soil temperature and moisture in the uppermost 20 cm were consistently measured from 23 June 2016 to 26 October 2016 using soil temperature-water monitors (L-99; Hangzhou Luge Science and Technology limited company, Hangzhou, China). The average temperature was 23.34 ± 1.80°C from 8:00 to 20:00 and 19.80 ± 0.31°C from 20:00 to 8:00 (Supplementary Figure S1). Therefore, to assess potential N_2_O emission rates we set the temperatures to range from 20.1 to 25.4°C from 8:00 to 20:00 and from 18.8 to 21.7°C from 20:00 to 8:00, which was consistent with the measured data. The soil moisture was set at a water-filled pore space (WFPS) of 60%, the wet soil environment which is conducive to N_2_O emission by denitrifiers (Bateman and Baggs, 2005; Li et al., 2016). Furthermore, the denitrification process occurs in anaerobic environments (Xiong et al., 2017) and thus an anaerobic environment was created using 150-ml Erlenmeyer flasks with gas-tight lids fitted with a gas sampling port and incubated an incubator (RXZ-380C; Ningbo Jiangnan Instrument Plant, Ningbo, China) (Bateman and Baggs, 2005). Soil (10 g, 7 repetitions, air-dried) was weighed into each flask and soil moisture amended to achieve the target WFPS of 60%. Soils were conditioned at 60% WFPS for 7 days to initiate microbial activity and to minimize changes in soil moisture in the incubator at the start of experiment. On day 8, the flasks were sealed, evacuated and filled with high argon gas 3 times (10 min each time) to keep an anaerobic environment. Samples were then incubated for 24 h and headspace samples (approximately 40 ml) were collected to assess for N_2_O gas. The N_2_O gas samples were analyzed using a gas chromatograph (Agilent 7890 gas chromatograph equipped with an ECD detector, Agilent, Santa Clara, CA, United States). The potential N_2_O emission rates were calculated using the following formula (Lu et al., 2011):

$$V_{N_{2}O}=\frac{\left[\rho \times C\times (V_{G}+V\times \alpha )\times 273\right]}{\left[W\times (273+T)\right]}$$

In this: *V*_N_2_O_ shows the potential N_2_O emission rates (mg kg^-1^ h^-1^); ρ shows the density of N_2_O–N in standard state, 1.25 kg m^-3^; *C* shows the gas density of N_2_O (m^3^ m^-3^); *V_G_* shows the upper effective volume of the flask (m^3^); *V* shows the liquid volume (m^3^); α shows the Bunsen correction coefficient, 0.549 in 25°C (we used the value in our study); *W* shows the dry soil weight (g); *T* is the temperature at the time gases were measured (we used a mean value in of 22°C).

#### Quantitative Polymerase Chain Reaction (Q-PCR) and Illumina MiSeq High-Throughput Sequencing

The soil DNA was extracted from 0.5 to 1 g soil using a D5625-01 soil DNA kit (Omega Biotek, Winooski, VT, United States) according to the manufacturer’s instructions. We extracted three soil DNA samples in each restoration stages for quantitative analysis, and then mixed the three DNA samples in one samples for Illumina MiSeq high-throughput sequencing analysis.

Quantitative analysis was conducted for fragments of the bacteria 16S rRNA and six denitrifying functional genes (i.e., *narG, napA, nirK, nirS, qnorB*, and *nosZ*). Q-PCR was performed in a CFX Real-Time PCR Detection System (Bio-Rad, Bio-Rad Laboratories Inc., Hercules, CA, United States) via a three-step thermal cycling procedure, with a 20 μL reaction mixture consisting of 10 μL of SYBR Green I PCR master mix (Applied Biosystems, Foster City, CA, United States), 1 μL of the DNA template (sample DNA or plasmid DNA for standard curves), 1 μL of forward primers, 1 μL of reverse primers, and 7 μL of sterile water (Millipore, Burlington, MA, United States). The protocol and parameter for each target gene are presented in Supplementary Table S4. The R-squared value for each standard curve exceeded 0.99, which indicated linear relationships across the ranges used in this study.

Successful PCR amplification was verified by 2% agarose gel electrophoresis. The triplicate amplicons were pooled and purified by gel extraction and quantified using a Quant-iT PicoGreen dsDNA Assay kit with a microplate reader (FLx800, BioTek Instruments, Inc., Winooski, VT, United States). The purified PCR amplicons were then mixed at equimolar ratios for sequencing analysis. Sequencing was conducted on the Illumina MiSeq platform using a TruSeq Nano DNA LT Library Prep Kit (Illumina Corporation, San Diego, CA, United States). Before sequencing, quality inspection was conducted on the Agilent Bioanalyzer using the Agilent High Sensitivity DNA Kit, and the sample was checked to have only a single peak. Then the sample was quantified using a Quant-iT PicoGreen dsDNA Assay Kit on a Promega QuantiFluor, and more than 2 nM was quantified. The sample was diluted and mixed with NaOH to denature the DNA into single strands for sequencing. Finally, the sample was analyzed using a MiSeq sequencer for paired-end sequencing of 2 × 300 bp using MiSeq Reagent Kit V3 (600 cycles).

#### Soil Physicochemical Analysis

The soil organic carbon and total nitrogen concentrations were determined using the oil bath-K_2_Cr_2_O_7_ titration method and the Kjeldahl method, respectively. Extractable ammonium and nitrate concentrations were determined following extraction of fresh soil with 1 mol L^-1^ KCl and then using a colorimetric method on an Alpkem Autoanalyzer (AA3 Auto Analyzer 3; German SEAL; German). The soil pH was determined using an automatic titrator (PHSJ-4F pH meter, Shanghai Electric Instrument Science Instruments Ltd., Shanghai, China) in 1:2.5 soil: water suspensions. The soil bulk density was determined gravimetrically in the laboratory.

### Statistical Analysis

To estimate the effects of *R. pseudoacacia* plantations on soil properties, potential N_2_O emission rates and denitrifying bacterial community, we analyzed the differences in soil bulk density, pH, organic carbon content, extractable ammonium and nitrate content, potential N_2_O emission rates, and the abundance of total bacteria and denitrifying functional genes (*napA, narG, nirK, nirS, qnorB*, and *nosZ*) with restoration age using general linear models. *Post hoc* comparisons with restoration stages were also performed using least significant difference (LSD) tests.

Principal Component Analysis (PCA) was used to analyze the similarity of total bacterial and denitrifying bacterial communities during restoration stages. The richness, evenness, and diversity of total bacterial communities were quantified by using Chao 1, Simpson and Shannon diversity indices, and the features of vegetation communities were quantified by using species richness, Pielou evenness and Shannon diversity indices. To quantify the correlations of potential N_2_O emission rates and denitrifying functional genes, we used a normal distribution of the log of abundance of denitrifying functional genes. The correlations among soil properties, potential N_2_O emission rates and denitrifying functional genes were quantified using Pearson correlation analyses before engaging in further analyses. Then, based on the results of Pearson correlation analyses, a multiple regression analysis was performed to determine the multiple linear regression equation relating potential N_2_O emission rates and denitrifying functional genes. Likewise, the structural equation model (SEM) was applied to investigate the direct and indirect effects of the indices chosen by multiple regression analysis on potential N_2_O emission rates (*P* > 0.05). Finally, PCA and Pearson correlation analyses were also used to determine the ordination of potential N_2_O emission rates, soil properties and denitrifying bacterial community (the three major denitrifiers in genes level of each denitrifying functional genes).

All statistical analyses were performed using SPSS (IBM SPSS Statistics 20.0; International Business Machines Corporation, Armonk, NY, United States), and all graphs were made using Origin (OriginPro 2016; OriginLab Corporation, Northampton, MA, United States).

## Results

### Potential N_2_O Emission Rates on Denitrification After a Short Incubation

**FIGURE 1:**
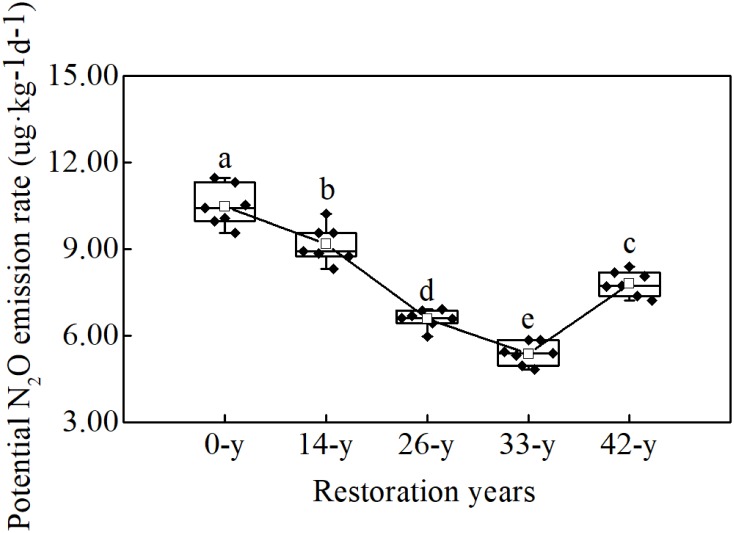
Different letters indicate significant differences (*P* < 0.05) among afforestation restoration ages based on a general linear model followed by an LSD *post hoc* test. The box plot shows the following information from top to bottom: maximum, upper quartile, median, lower quartile, and minimum. The white square shows the average value and the black line shows the trend. The black rhombus shows potential N_2_O emission rate data.

Potential N_2_O emission rates varied significantly with restoration ages and ranged between 5.38 and 10.50 μg kg^-1^ d^-1^ (Figure 1; *df* = 4; *F* = 107.313; *P* < 0.000). Rates were the highest at the 0-year sites, and decreased as the restoration ages increased from the 14-year to 33-year sites, and then increased between the 33-year and 42-year sites.

### Abundance of Denitrifying Functional Genes

**FIGURE 2:**
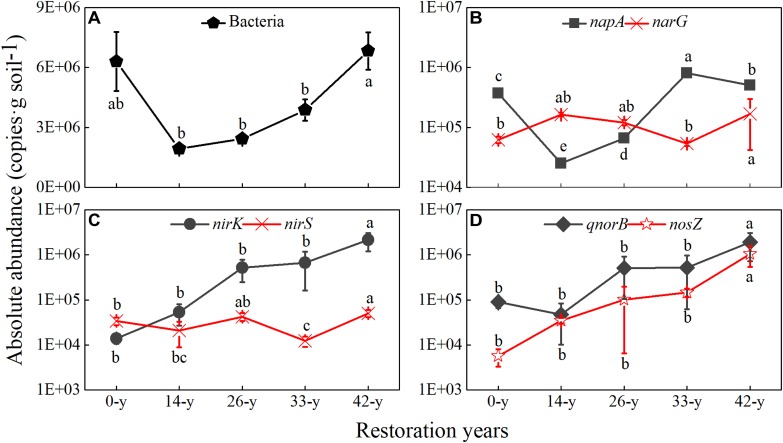
The absolute abundances of total bacteria and denitrifying functional genes among afforestation restoration of abandoned farmland. **(A)** Bacteria; **(B)** *napA, narG*; **(C)** *nirK, nirS*; **(D)** *qnorB, nosZ*. The absolute abundances (copies g soil^-1^) are shown on a log 10 scale (Y-axis). The standard deviations of the three replicates are indicated by error bars. Non-visible error bars indicate that the standard deviations are smaller than the marker size. Different letters indicate significant differences (*P* < 0.05) among restoration ages based on a general linear model followed by an LSD *post hoc* test.

The absolute abundances of total bacteria and denitrifying functional genes, except *narG* genes, varied significantly with restoration stage (Figure 2 and Supplementary Table S5). The absolute abundances of total bacteria ranged from 1.93 × 10^6^ to 6.82 × 10^6^ copies g^-1^, and initially decreased compared with the 0-year sites and subsequently increased at the 14-year - 42-year sites. The *napA* and *narG* genes exhibited different trends. The absolute abundances of *napA* and *narG* genes varied 2.51 × 10^4^ - 8.11 × 10^5^ copies g^-1^ and 5.35 × 10^4^ - 1.70 × 10^5^ copies g^-1^, respectively. Compared with *narG* gene, which showed no obvious trend across the sites, the *napA* gene initially decreased between 0-year and 14-year sites, and subsequently increased at the 14-year - 33-year sites, and then declined between the 33-year and 42-year sites. The absolute abundances of *nirK, qnorB* and *nosZ* genes appeared to be more abundant at the 42-year sites than at those of other sites and increased from the 0-year to 42-year sites. In contrast, the *nirS* gene abundances showed no obvious trend across sites. The *nirK, nirS, qnorB*, and *nosZ* genes varied 2.8 × 10^3^–7.59 × 10^5^, 2.58 × 10^3^-9.77 × 10^3^, 1.90 × 10^4^-9.61 × 10^5^, and 1.94 × 10^3^-4.20 × 10^5^ copies g^-1^, respectively. Furthermore, the absolute abundances of *qnorB* were more than those of *nosZ* in all sites.

### Diversity and Composition of Total Bacterial and Denitrifying Bacterial Communities

After quality trimming and chimera removal, the total bacterial and denitrifying bacterial sequences were assayed using Illumina MiSeq high-throughput sequencing (Supplementary Table S6). The variations of total bacteria and denitrifying bacteria composition with restoration stages were identified by PCA (Supplementary Figures S2, S3). The richness (shown by Chao 1 index) and diversity (shown by Shannon index) of the total bacterial communities revealed the similar trend, i.e., an initial increase followed by a decrease (except 42-year in richness), with the highest values at the 26-year sites (Supplementary Table S7). However, the evenness (shown by Simpson index) of the total bacterial communities showed no difference among restoration stages (Supplementary Table S7). Furthermore, the richness, evenness and diversity of denitrifying bacterial communities showed no obvious trends among restoration stages (Supplementary Table S7). The total bacterial community was dominated by *Actinobacteria* (23.1% on average), followed by *Proteobacteria* (21.7%), *Acidobacteria* (17.1%), *Planctomycetes* (12.8%), *Gemmatimonadetes* (7.2%), and *Chloroflexi* (7.2%) (with relative abundances of more than 5%; Supplementary Table S8). *Proteobacteria* was the major phylum in total bacterial communities at 0-year to 33-year sites. However, *Acidobacteria* and *Planctomycetes* were dominant at 42-year sites (Supplementary Table S9). Moreover, the denitrifying bacteria encoded by *napA, narG, nirS*, and *nosZ* genes were dominated by *Proteobacteria* in the phyla level, and the percentages of *Proteobacteria* in denitrifying bacteria communities were 47.8, 56.0, 5.8, and 89.8%, respectively. *Actinobacteria* and *Proteobacteria* were the major phyla of denitrifying bacteria encoded by *nirK* (16.8%, 15.7%) and *qnorB* (8.9%, 12.8%), but the denitrifying bacterial communities were still somewhat unclear.

### Soil Properties, Abundance and Diversity of Denitrifying Bacterial Communities in Relation to the Potential N_2_O Emission Rates

**FIGURE 3:**
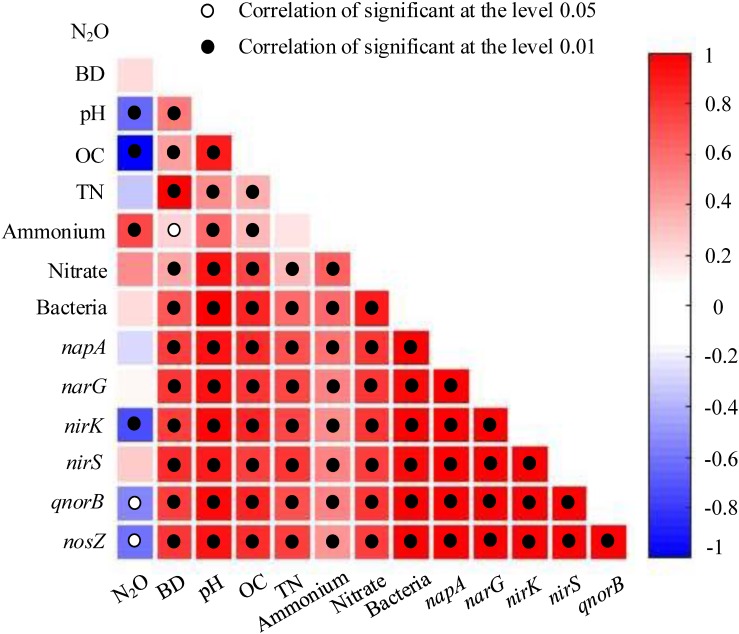
The relationships between potential N_2_O emission rates, denitrifying functional gene abundances, and soil properties among restoration stages. The strength of the linear relationship between two variables is described by the Pearson Correlation Coefficient and showed in different colors. *P*-values are given through black circles (*P* < 0.01) and unfilled circles (*P* < 0.05). N_2_O = N_2_O emission rates (μg kg^-1^ d^-1^); BD = bulk density (g cm^-1^); OC = organic carbon (g kg^-1^); TN = total nitrogen (g kg^-1^); extractable ammonium and nitrate (mg kg^-1^); bacteria, *napA, narG, nirK, nirS, qnorB, and nosZ* show the abundances of denitrifying functional genes.

**FIGURE 4:**
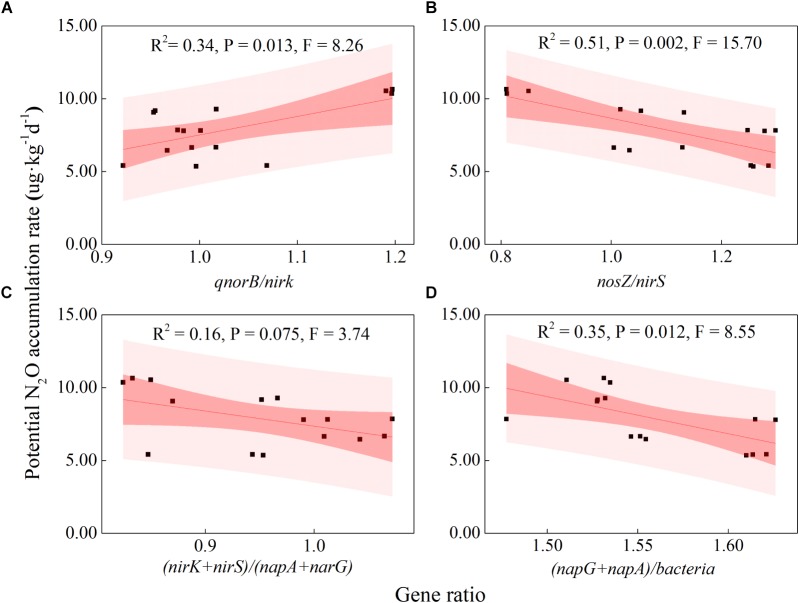
Relationships between the potential N_2_O emission rates and denitrifying functional gene ratios across restoration stages. **(A)** *qnorB/nirK* (NO transformation and N_2_O production); **(B)** *nosZ/nirS* (NO to N_2_ transformation); **(C)** (*nirK+nirS*)/ (*napA+narG*) (nitrite consumption); **(D)** (*napA+narG*)/*bacteria* (nitrite production). The bacteria represent the abundance of the denitrifier bacterial 16S RNA gene. The black squares indicate the potential N_2_O emission rates and the ratios of denitrifying functional genes. The red fitted lines are from ordinary least squares regression. The dark and shaded areas show the 95% confidence interval and the prediction band of the fit, respectively.

**FIGURE 5:**
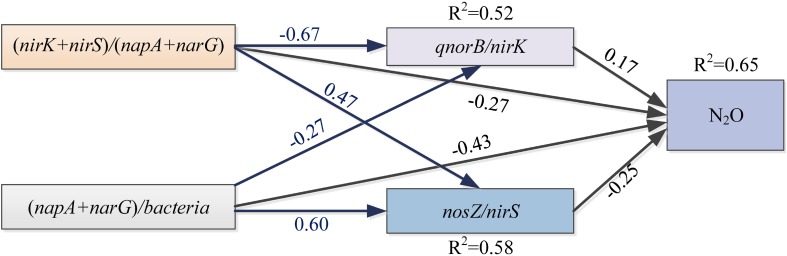
The structural equation model (SEM) describing the direct and indirect contributions of gene fragments on the potential N_2_O emission rates. Single-pointed arrows indicate causal paths. The black arrows represent the direct influences, and the blue arrows show indirect influences. *R*^2^ values are shown for dependent variables. A non-significant *P*-value (*P* = 0.086) for the Chi-squared statistic indicate that there was no significant difference between the covariance pattern predicted by the SEM and that from the observed covariance, indicating a good fit of the data.

Some soil properties and soil microbial abundances were correlated with potential N_2_O emission rates. Soil pH and organic carbon content were negatively related to potential N_2_O emission rates (Figure 3; *P* < 0.01), while a positive relationship was found between extractable ammonium content and potential N_2_O emission rates (Figure 3; *P* < 0.01). Furthermore, the abundances of denitrifying bacteria encoded by *nirK, qnorB*, and *nosZ* genes were negatively related to potential N_2_O emission rates (Figure 3; *P* < 0.01, *P* < 0.05, and *P* < 0.05, respectively). Multiple regression analysis was used to determine the relative contribution of denitrifying functional genes to the variation of potential N_2_O emission rates. As a result, the ratios of *qnorB/nirK, nosZ/nirS*, (*nirK*+ *nirS*)/(*napA*+ *narG*), and (*napA*+ *narG*)/*bacteria* were the four major predictors, which could explain 53% of the variation in potential N_2_O emission rates (*P* = 0.018). The ratios of *qnorB*/*nirK, nosZ*/*nirS*, and (*napA*+ *narG*)/*bacteria* were significantly explained 34, 51, and 35% of the variation in potential N_2_O accumulation rates, respectively (Figure 4A,B,D). However, the ratios of (*nirK*+ *nirS*)/(*napA*+ *narG*) had no significant influence on potential N_2_O emission rates (Figure 4C). Likewise, the *qnorB*/*nirK, nosZ/nirS*, and (*nirK*+ *nirS*)/(*napA*+ *narG*) ratios showed significant differences between the 0-year and 14-year - 42-year sites (Supplementary Figures S4a–c), and (*napA*+ *narG*)/*bacteria* ratios were significantly different between the sites with the lowest (0-year) and the highest (33-year) potential N_2_O emission rates (Supplementary Figure S4d). Then, we used a SEM to examine the direct and indirect effects of the ratios of denitrifying functional genes on the potential emission rates of N_2_O (Figure 5). The *qnorB*/*nirK, nosZ*/*nirS*, (*nirK*+ *nirS*)/(*napA*+ *narG*), and (*napA*+ *narG*)/*bacteria* ratios had direct different influences (β = 0.17,-0.25, -0.27, and -0.43) on the variation of the potential N_2_O emission rates. Furthermore, the influences of (*nirK*+ *nirS*)/(*napA*+ *narG*) on the variation of the potential emission rates of N_2_O were mediated through *qnorB*/*nirK* (β = -0.11) and *nosZ/nirS* (β = -0.12), and the (*napA*+ *narG*)/*bacteria* ratios affected the potential N_2_O emission rates indirectly through *qnorB/nirK* (β = -0.04) and *nosZ/nirS* (β = -0.15). Overall, the SEM had a *P* = 0.086 (*P* > 0.05), which indicated that the SEM was applicable and explained 65% of the total variance influencing the potential N_2_O emission rates.

**FIGURE 6:**
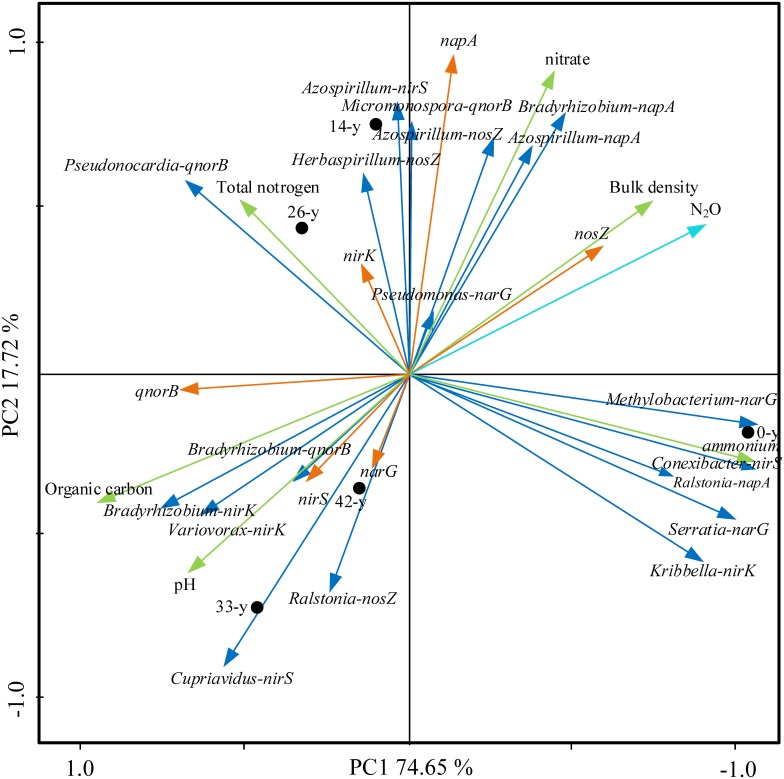
Principal component analysis (PCA) of potential N_2_O emission rates, soil properties (bulk density, pH, organic carbon content, total nitrogen content, extractable ammonium and nitrate content, the abundances (showing by *napA, narG, nirK, nirS, qnorB*, and *nosZ*) and composition of denitrifying bacterial community (*Bradyrhizobium-napA, Azospirillum-napA, Ralstonia-napA, Methylobacterium-narG, Serratia-narG, Pseudomonas-narG, Kribbella-nirK, Bradyrhizobium-nirK, Variovorax-nirK, Conexibacter-nirS, Azospirillum-nirS, Cupriavidus-nirS, Micromonospora-qnorB, Pseudonocardia-qnorB, Bradyrhizobium-qnorB, Azospirillum-nosZ, Ralstonia-nosZ*, and *Herbaspirillum-nosZ*). The first two PCA axes (PC1 and PC2) explain 92.37% of total variance. Field plots coded as in Supplementary Table S2.

Ordination of samples by PCA based on potential N_2_O emission rates, soil properties, abundances and composition of denitrifying bacterial community showed a clear separation of restoration stages along the first axis, with the first two axes explaining 92.37% of the total variance (Figure 6). The ordination stressed the trends observed in Supplementary Table S11, showing a strong correlation of some soil properties, denitrifying bacterial community with potential N_2_O emission rates. Of particular interest, we found positive correlations among extractable ammonium content, the abundance of *nosZ* and *napA* and potential N_2_O emission rates (*P* < 0.01), while negative correlations among soil pH, organic carbon content, the abundances of *narG, nirS* and *qnorB, Bradyrhizobium* (*nirK*), *Variovorax* (*nirK*), *Bradyrhizobium* (*qnorB*), and potential N_2_O emission rates were found (*P* < 0.01).

## Discussion

### Potential Rates of N_2_O Emission Across a Restoration Chronosequence

The potential N_2_O emission rates through denitrification process varied significantly with restoration ages (Figure 1), suggesting that afforestation restoration does indeed alter N_2_O emissions (Liu et al., 2018). Compared with the 14-year - 42-year sites in this study, the 0-year sites had by far the highest potential N_2_O emission rates, indicating that farmland may have been the larger source of N_2_O (Domeignoz-Horta et al., 2018). Unfortunately we cannot scale these potential rates to annual values; nevertheless, elevated rates in actively managed farmlands would not be a surprise. Agricultural practices, such as tillage and fertilization, can increase the soil organic carbon and extractable ammonium contents consequently alter N_2_O emissions (Suleiman et al., 2013). Furthermore, afforestation restoration rapidly created a specific understory microhabitat due to canopy density, showing a decreasing trend of soil potential N_2_O emission (except at the 42-year sites; Figure 1). This supports the hypothesis that restoration in soils with a higher pH, such as those common in arid environments, may reduce N_2_O emission (Gundersen et al., 2012). Šimek et al. (2002) also reported that N_2_O/(N_2_O + N_2_) ratios decreased with increasing soil pH values, consistent with our finding that soil potential N_2_O emission rates decreased and pH increased at 14-year - 42-year sites (Figure 1 and Table 1). The 42-year sites had a higher potential N_2_O emission rates than did the 33-year sites (Figure 1), following a decrease in soil pH and organic carbon content, which translated into a decline in ecological function in the oldest sites. This phenomenon was consistent with the finding by Kou et al. (2016), who found that the soil nutrients and the density and cover of *R. pseudoacacia* plantations decreased after 30 years of afforestation in the Chinese Loess Plateau. Understory vegetation communities can dramatically alter soil microbial process through root exudation and litter inputs (Singh et al., 2004). In this study, we found that the potential fluxes of N_2_O were negatively correlated with trends in understory vegetation features, such as richness, evenness and diversity (Supplementary Table S3), and that soil properties, such as soil pH and organic carbon content (Table 1), were also negatively correlated with potential N_2_O emission rates, perhaps through their interactions with changes in understory vegetation (Cuhel et al., 2010; Levy-Booth et al., 2014). Therefore, N_2_O emission can be influenced by understory vegetation, soil pH and organic carbon content over the course afforestation and the role of denitrifying bacteria may also be critical.

### The Abundance Dynamic of Denitrifying Functional Genes During Afforestation Restoration

The absolute abundances of total bacteria and the denitrifying functional genes of *nirK, qnorB*, and *nosZ* increased across age since restoration plantings (Figure 2), suggesting that afforestation restoration can increase microbial biomass (Lozano et al., 2014; Barber et al., 2017). However, the absolute abundances of total bacteria and the *qnorB* gene in the 0-year sites were higher than in the 14-year sites (Figure 2), which was inconsistent with a previous study showing that frequent human intervention can reduce the abundance of bacteria (Lin et al., 2012). Added nutrient resources in farmlands may support a more plentiful microbial community (Zhang C. et al., 2016), an idea which was supported by the Shannon diversity of the total bacterial community (Supplementary Table S5). Organic carbon content was positively correlated with the abundance of *napA* and *narG* genes (Bru et al., 2011). In this study, the absolute abundances of *napA* and *narG* genes showed different changes with restoration stages (Figure 2B), which may be explained by the special roles of *napA* and *narG* genes (Chèneby et al., 2009). The *napA* genes had a similar change with soil pH and organic carbon content (Figure 2B and Table 1), suggesting that the *napA* gene is more sensitive than *narG* gene on the variations in soil nutrients, which is contrary to the finding by Chèneby et al. (2009). The *narG* gene showed no obvious change with restoration age, inconsistent with the finding that the *narG* gene is easily promoted by increases in soil nutrients (Tang et al., 2016). This discrepancy might be due to differences in the environmental conditions in the two studies. The Chinese Loess Plateau is located in a semiarid region, whereas the *nirS* gene is likely more adapted to waterlogged soils than the *nirK* gene (Azziz et al., 2017). The *nirS* genes also showed no obvious changes in absolute abundance, while the *nirK* gene increased among restoration ages (Figure 2C). Likewise, the *qnorB* genes had higher abundances than *nosZ* genes in all sites (Figure 2D), which offered a molecular-level explanation for the viewpoint that soil acts as a source for N_2_O, especially in farmlands and forests (Bremner and Blackmer, 1979; Kong et al., 2010; Levy-Booth et al., 2014; Hu et al., 2015). Briefly, denitrifying functional genes have different roles in denitrification and have close relationships with each other (Figure 3), which can change the products of denitrification, especially N_2_O emission (Hu et al., 2015).

### Variation in Denitrifying Bacterial Community Over the Course of Afforestation Restoration

PCA of the compositions and structures of the total bacterial and denitrifying bacterial communities identified large shifts in the distributions of microbial communities during restoration stages (Supplementary Figures S2, S3), which was consistent with the results of Lozano et al. (2014) and Zhang C. et al. (2016), who found obvious variations during the succession of bacterial communities in abandoned farmland. *Actinobacteria* and *Proteobacteria* were the most two abundant phyla of the total bacterial communities regardless of the restoration stages (except 42-year sites; Supplementary Table S8). Soil nutrients play an important role in the total bacterial community composition for *Actinobacteria* can be widely distributed in both terrestrial and aquatic ecosystems (Ventura et al., 2007) and *Proteobacteria* is partial to nutrient-rich soils (Liu et al., 2018). In this study, the total bacterial communities transitioned from *Actinobacteria*-dominant (0-year) to *Proteobacteria*-dominant (14 and 26-year) and then returned to *Actinobacteria*-dominant (33-year) (Supplementary Table S9), consistent with the findings by Desnues et al. (2007) and Liu et al. (2018). Taken together, these results indicate that total bacterial community composition was more influenced by soil total nitrogen and extractable nitrate contents. Furthermore, we found that the highest richness, evenness, and diversity of the understory vegetation community were in the 33-year sites, while the highest richness and diversity of total bacterial community were in the 26-year sites (Supplementary Tables S3, S7), suggesting an incongruous process which understory vegetation succession followed soil bacteria succession. These patterns are inconsistent with Lozano et al. (2014), who found that microbial succession followed plant succession in natural recovery processes. The mechanism underlying this difference is unclear, and the effects of artificial restoration process, especially for *R. pseudoacacia* plantations, on soil ecosystem may aid in understanding. Specifically, the total bacterial community composition in 42-y sites was dominated by *Planctomycetes* (22.3%), followed by *Actinobacteria* (19.4%), *Acidobacteria* (18.7%), and *Proteobacteria* (14.4%) (Supplementary Table S9), indicating a unique distribution of total bacterial community composition was found in our study. The mechanism underlying this control is unclear, but vegetation community in the herbaceous layer and the limitation by soil nutrients are likely playing a role.

Furthermore, *Actinobacteria* and *Proteobacteria* were the major phyla of denitrifying bacteria encoded by *nirK* and *qnorB* genes, which indicated that N_2_O products can occur in most environments (Liu et al., 2018) Although other soil processes controlled by microbes, such as nitrification, can also contribute to N_2_O efflux, denitrification is often a dominant pathway. *Proteobacteria* was the major phylum of denitrifying bacteria encoded by *napA, narG, nirS*, and *nosZ* regardless of restoration stages (Supplementary Table S3), suggesting that the dynamics of N_2_O emission is influenced by the changes in soil nutrients (Azziz et al., 2017; Tatti et al., 2017), which is in agreement with the relationship analysis indicating that denitrifying functional genes and soil properties were closely related (Figure 3). However, the richness, evenness, and diversity of denitrifying bacterial community showed no obvious trends across the chronosequence (Supplementary Table S7). The mechanism underlying this is unclear.

### Relationships Among Potential N_2_O Emission Rates, Soil Properties and Denitrifying Bacteria

Taken together, the correlation analyses and the PCA (Figure 6) suggested that the variation in potential N_2_O emission rates were partially attributable to changes in the soil properties and in the abundance and composition of denitrifying bacteria. In addition, the restoration occurring from afforestation studied here changed the canopy density and altered the understory community (Liu et al., 2018). Such changes to the vegetation can affect soil properties through changes to root exudation and litterfall (Singh et al., 2004).

#### Response of Potential N_2_O Emission Rates to Soil Properties

Afforestation via planting trees can rapidly create an understory microhabitat from increases canopy density, which can, in turn, alter soil properties and N_2_O fluxes (Liu et al., 2018). In our study, we found that soil extractable ammonium content, pH, organic carbon content, and bulk density all had close correlations with potential N_2_O emission rates (Figure 3, 6). Specifically, we found a strong positive correlation between extractable ammonium content and potential N_2_O emission rates (Figure 3). Our study area was farmlands that were fertilized and agriculturally managed, especially with fertilizer addition, which can supply ample ammonium for denitrification, and N_2_O emission (Hu et al., 2015). Furthermore, soil pH had a negative correlation with potential N_2_O emission rates (Figure 3, 6 and Supplementary Table S11), consistent with the findings that higher soil pH could reduce N_2_O efflux (Wrage et al., 2001; Hu et al., 2015). Our results indicated that the denitrifying bacterial community encoded by *nosZ* genes preferred low pH in soils, which is seen in the negative relationships between soil pH and the Shannon diversity of denitrifying bacterial community encoded by *nosZ* genes (Figure 6). Soil organic carbon serves as the energy supply in heterotrophic microbial pathways, yet soil carbon content was negatively correlated with potential N_2_O emission rates (Figure 3, 6 and Supplementary Table S11). These patterns may be explained by the denitrifying bacterial community in the phyla level. We found that denitrifying bacterial community encoded by *qnorB* genes, which can produce N_2_O, belongs to *Actinobacteria* and *Proteobacteria*, leading to a large number of N_2_O emission in soils (Liu et al., 2018). However, the *nosZ* genes, acting as the only microbial pathway for N_2_O consumption, were made up more by *Proteobacteria*, meaning that eutrophic environments can had lower N_2_O emissions (Hu et al., 2015). Soil bulk density, which can be related to soil aeration, was positively correlation with potential N_2_O emission rates (Figure 6), suggesting that soil compaction could represent a significant control over N_2_O emission rates and losses to the atmosphere (Hu et al., 2015). Therefore, fertilizer addition and soil compaction (i.e., higher soil bulk density) may increase N_2_O emissions, whereas higher soil pH and organic carbon content can reduce N_2_O emissions.

#### Response of Potential N_2_O Emission Rates to Denitrifying Functional Genes

The multiple regression analysis, SEM and PCA showed that total bacteria and denitrifying functional genes were crucial in mediating potential N_2_O emission rates (Supplementary Table S10 and Figure 5), which could reflect and integrate a part of the fluctuations in bio-ecological processes (Hu et al., 2015). More specifically, the *qnorB/nirK* denotes the level of NO transformation and N_2_O production as the *qnorB* genes are directly involved in N_2_O production and NO consumption, while *nirK* genes perform NO production. The significant positive correlation between *qnorB*/*nirK* and potential N_2_O emission rates indicated the importance of the reaction substrate supply in N_2_O emissions. The *nirK* and *nosZ* genes are directly involved in NO production and N_2_ production processes, and *nosZ*/*nirS* ratio reflects the level of NO to N_2_ transformation. The *nosZ*/*nirS* had a negative correlation with potential N_2_O emission rates and because N_2_ production is the only pathway for N_2_O consumption, these data suggested that *nosZ* genes play an important role in controlling N_2_O emissions for this system. The *napA* and *narG* genes are involved in nitrite consumption level in denitrification process. The (*napA*+ *narG*)/*bacteria* and (*nirK*+ *nirS*)/(*napA*+ *narG*) were negatively associated with potential N_2_O emission rates, likely because they reflect nitrite transformation in the denitrification process. (*napA*+ *narG*)/*bacteria* reflects the nitrite production level and (*nirK*+ *nirS*)/(*napA*+ *narG*) was directly involved in nitrite consumption. This is in agreement with the finding of Levy-Booth et al. (2014), who found that nitrite reduction had a major influence on N_2_O emission rates. Furthermore, the indirect influence of denitrifying functional genes on potential N_2_O emission rates reflects the close links among different microbial processes of denitrification. The ratios of (*nirK*+ *nirS*)/(*napA*+ *narG*) and (*napA*+ *narG*)/*bacteria* had indirect influences on potential N_2_O emission rates through *qnorB*/*nirK*, suggesting that nitrite transformation showed an indirect effect on potential N_2_O emission rates by influencing the N_2_O production. We also found that the ratios of (*nirK*+ *nirS*)/(*napA*+ *narG*) and (*napA*+ *narG*)/*bacteria* had indirect influences on potential N_2_O emission rates through *nosZ/nirS*, suggesting that nitrite transformation showed an indirect effect on N_2_O emission rates by influencing the N_2_O consumption. Notably, we also found that the abundances of denitrifying bacteria encoded by *nosZ* were positively correlated with potential N_2_O emission rates, while *qnorB* was negatively correlated with potential N_2_O emission rates. The *qnorB*/*nosZ* ratios were positively correlated with potential N_2_O emission rates (*r* = 0.41), indicating that denitrifying functional genes have close relationships with each other (Figure 3) and N_2_O emissions can be regulated by the abundance of denitrifying functional genes (Hu et al., 2015).

Therefore, nitrite transformation is a key step in denitrification process and N_2_O emission (Domeignoz-Horta et al., 2018). However, the relationships between denitrifying functional genes and potential N_2_O emission rates were not sufficient to fully explain patterns in N_2_O emissions, and the underlying mechanisms and the response of denitrifying bacterial community on N_2_O emission rates is worthy of further study.

#### Response of Potential N_2_O Emission Rates to Denitrifying Bacterial Community

N_2_O emission is driven by soil microorganisms, especially denitrifiers in anaerobic environments (Hu et al., 2015). In our study, we found *Bradyrhizobium* (*nirK*) and *Variovorax* (*nirK*) were negatively correlated with potential N_2_O emission rates (*P* < 0.01) (Figure 6 and Supplementary Table S11), suggesting that the denitrifying functional genes encoded by *nirK*, which produce NO, were crucial to N_2_O emission. These data point to nitrite transformation as a key step in determining overall N_2_O emission rates, at least under the conditions simulated here (found in section “Response of Potential N_2_O Emission Rates to Denitrifying Functional Genes”). Specifically, a negative correlation between *Bradyrhizobium* (*qnorB*) and potential N_2_O emission rates was found, inconsistent with the finding that denitrifiers encoded by *qnorB* can produce N_2_O. In our study, soil organic carbon content had the strongest relationship with potential N_2_O emission rates, followed by the abundance of *nosZ*, soil bulk density, and *Bradyrhizobium* and *Variovorax* abundance, consistent with the findings by Levy-Booth et al. (2014), Lozano et al. (2014), Zhang C. et al. (2016), and Domeignoz-Horta et al. (2018), which indicated that the changes of soil properties can alter the microorganism composition and then influence the products of soil microbial pathway. Taken together, our study and others emphasize the importance of denitrifying bacterial communities in responding to environmental changes and modulating denitrification process (Bernhard and Kelly, 2016).

## Conclusion

Afforestation restoration rapidly created a special understory microhabitat that acted as driving force to alter N_2_O emission and the diversities and compositions of vegetation and soil denitrifying bacteria communities. Compared with farmlands, afforestation restoration can increase soil nutrients (soil organic carbon content) and improve soil physical characteristics (soil bulk density), followed by increasing the abundances of denitrifying functional genes and altering the structure of denitrifying bacterial communities (*Actinobacteria*-dominant to *Proteobacteria*-dominant). These changes can interact to alter the dynamics of potential N_2_O emission rates. Specifically, correlation analysis indicated that NO transformation was the key step in determining potential N_2_O emissions in our study. However, some denitrifying bacteria were remaining unknown, and these organisms could be targeted for future soil nitrogen-focused studies in semiarid ecosystems.

## Author Contributions

ND made the text, figures, and tables (including the Supplementary Material). HW, SH, and JJ contributed to experimental design method, manuscript frame, and manuscript modification.

## Conflict of Interest Statement

The authors declare that the research was conducted in the absence of any commercial or financial relationships that could be construed as a potential conflict of interest.
