# Supplementary material for: Effects of Afforestation Restoration on Soil Potential N2O Emission and Denitrifying Bacteria After Farmland Abandonment in the Chinese Loess Plateau
Source: Front Microbiol. 2019 Feb 19;10:262. doi: 10.3389/fmicb.2019.00262 (PMC6389719; doi:10.3389/fmicb.2019.00262)
Supplement: Supplementary file 1 [file Data_Sheet_1.docx]

**Frontiers in microbiology**

**Effects of artificial restoration on N_2_O emission after farmland abandonment in the Chinese hilly-gullied Loess Plateau**

**Na Deng, Honglei Wang*, Shu Hu, Juying Jiao***

State Key Laboratory of Soil Erosion and Dry Land Farming on the Loess Plateau, Institute of Soil and Water Conservation, Northwest A & F University, Yangling 712100, Shaanxi, China.

***Correspondence to: Honglei Wang and Juying Jiao,** Institute of Soil and Water Conservation, Northwest A & F University, No. 26, Xinong Road, Institute of Soil and Water Conservation, Yangling, Shaanxi 712100, PR China. Email: wanghonglei@nwsuaf.edu.cn and [jyjiao@ms.iswc.ac.cn](mailto:jyjiao@ms.iswc.ac.cn)





**Fig. S1** The green and blue polylines show the trend of soil temperature and moisture, and the pink and blue straight line show the averages of soil temperature and moisture.

**Fig. S2** Ordinations based on principal component analysis and hierarchical cluster analysis to evaluate the variations of bacteria communities during 42 years artificial restoration. The first two PCA axes explain 52.38% of total variance, respectively. Different numbers adjacent to arrows represent the relative distance between soil sites.

**Fig. S3** Ordinations based on principal component analysis and hierarchical cluster analysis to evaluate the variations of denitrifying communities during 42 years artificial restoration. The first two PCA axes explain77.53%, 93.92%, 65.86%, 91.85%, 74.83% and 90.28% of total variance, respectively. Different numbers adjacent to arrows represent the relative distance between soil sites.


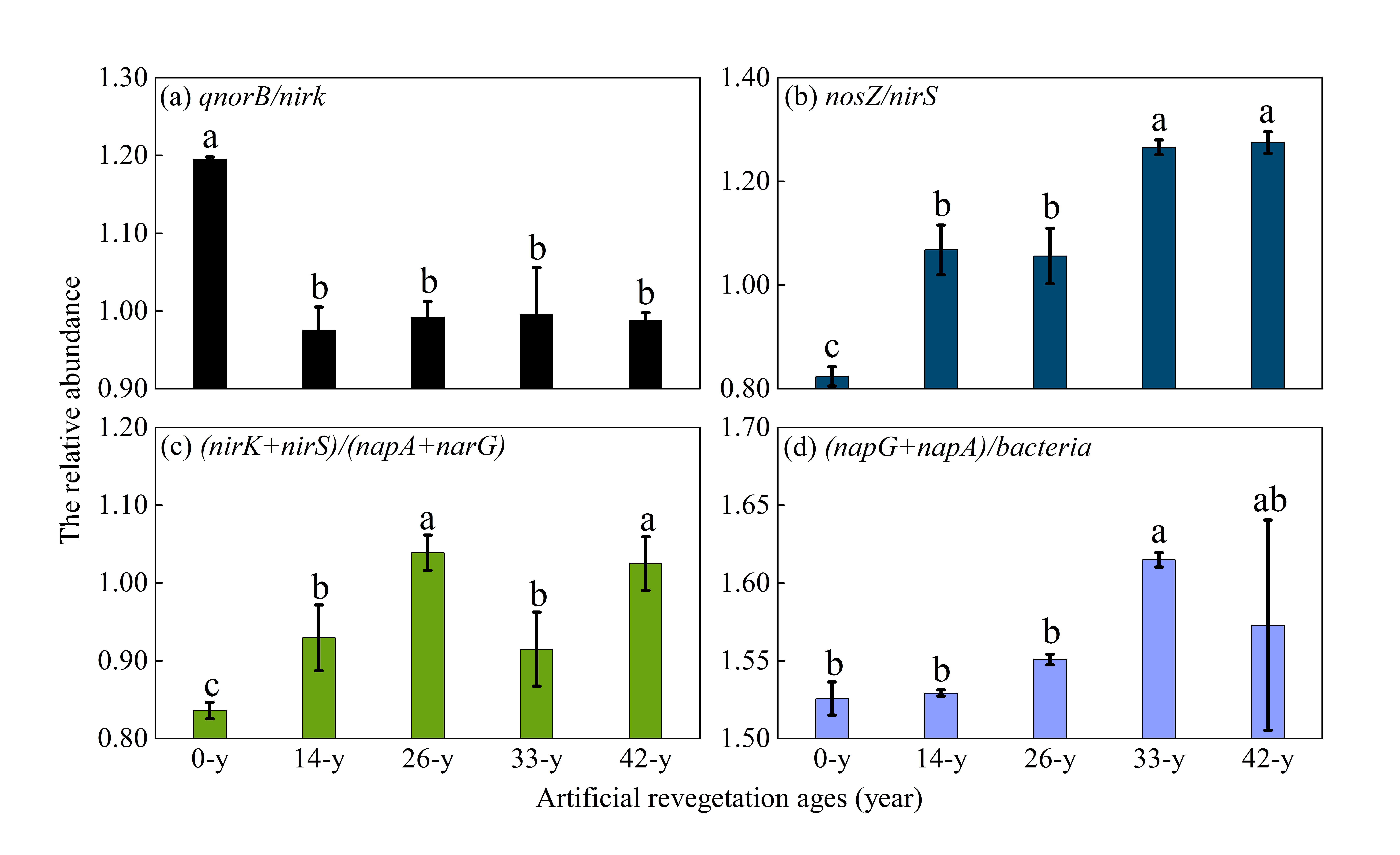


**Fig. S4** The relative abundances of the ratios of denitrifier bacteria and functional genes during artificial restoration from abandoned farmland. (a) *qnorB/nirK*; (b) *nosZ/nirS*; (c) *(nirK+nirS) /(napA+narG)*; (D) *(napA+narG)*/bacteria. The standard deviations are indicated by error bars. Invisible error bars indicate that the standard deviations are smaller than the marker size. Different letters indicate significant differences (P ˂ 0.05) during artificial restoration years based on a general linear model followed by an LSD test.

**Table S1** Variance analysis data of soil properties in *Robinia pseudoacacia* plantations sites.

| Index | *df* | F | P | Adjusted R^2^ |
| --- | --- | --- | --- | --- |
| BD | 4 | 78.125 | 0.000 | 0.969 |
| pH | 4 | 31.556 | 0.000 | 0.927 |
| OC | 4 | 6799.745 | 0.000 | 1.000 |
| TN | 4 | 1011.673 | 0.000 | 0.998 |
| Ammonium | 4 | 2772.711 | 0.000 | 0.999 |
| Nitrate | 4 | 110.841 | 0.000 | 0.978 |

Variance analysis was used a general linear model followed by an LSD test through SPSS 20.0. BD = bulk density (g·cm^-1^); OC = organic carbon (g·kg^-1^); TN = total nitrogen (g·kg^-1^); ammonium and nitrate (mg·kg^-1^); *df* = degree of freedom; F = F − value; P = P − value; Adjusted R^2^ = adjusted R-squared value.

**Table S2** The vegetation features of the *Robinia pseudoacacia* plantations sites.

| Sites | Slope(°) | Canopy density (%) | Coverage (%) | Understory vegetation species |
| --- | --- | --- | --- | --- |
| 0-y | - | - | - | - |
| 14-y | 30-38 | 37-52 | 30-55 | *Setaria viridis*, *Salsola collina*, *Bidens parviflora* |
| 26-y | 25-31 | 33-53 | 30-60 | *Artemisia gmelinii*, *Stipa bungeana* |
| 33-y | 26-31 | 57-73 | 50-80 | *Stipa bungeana*, *Solanum septemlobum*, *Melica scabrosa* |
| 42-y | 20-39 | 40-48 | 35-50 | *Stipa bungeana*, *Artemisia argyi*, *Artemisia gmelinii* |

The canopy density for *Robinia pseudoacacia* plantation; the coverage for understory vegetation species.

**Table S3** The features of vegetation communities in *Robinia pseudoacacia* plantations sites.

| Sites | Species richness | Pielou index | Shannon index |
| --- | --- | --- | --- |
| 14-y | 8 | 0.71 | 1.38 |
| 26-y | 12 | 0.72 | 1.47 |
| 33-y | 14 | 0.80 | 1.61 |
| 42-y | 13 | 0.61 | 1.58 |

Species richnesses show the richness, Pielou index show the evenness and Shannon index show the diversity of the vegetation communities.

**Table S4** Primers and thermal profiles used for real-time PCR quantification of the different phylogenetic and functional genes.

| Target gene | Primers | Primer sequence (5’-3’) | Thermal profile | Reference |
| --- | --- | --- | --- | --- |
| Bacteria 16S rRNA | 520F | AYTGGGYDTAAAGNG | 95°C 5 min; 95°C 60 s, 53°C 30 s, 72°C 60 s; 35 cycles | (Mahnert et al., 2015) |
|  | 802R | TACNVGGGTATCTAATCC |  |  |
| *napA* | napA V17F | TGGACVATGGGYTTYAAYC | 95°C 10 min, 95°C 15 s, 56.5°C 45 s, 72°C 30 s; 40cycles | (Bru et al., 2007) |
|  | napA 4R | ACYTCRCGHGCVGTRCCRCA |  |  |
| *narG* | narG-1960m2f | TA(CT)GT(GC)GGGCAGGA(AG)AAACTG | 95°C 10 min; 95°C 15 s, 58 °C 45 s, 72°C 30 s; 40cycles | (López-Gutiérrez et al., 2004) |
|  | narG-2050m2r | CGTAGAAGAAGCTGGTGCTGTT |  |  |
| *nirK* | nirK583F | TCA TGGTGCTGCCGCGKGACGG | 95°C 10 min; 95°C 15 s, 64°C 45 s, 72°C 30 s; 40cycles | (Yan et al., 2003) |
|  | nirK909R | GAA CTTGCCGGTKGCCCAGAC |  |  |
| *nirS* | *nirS*cd3aF | GT(C, G)AACGT(C, G)AAGGA(A, G)AC(C, G)GG | 95°C 10 min; 95°C 15 s, 64°C 45 s, 72°C 30 s; 40cycles | (Throbäck et al., 2004) |
|  | *nirS*R3cd | GA(C, G)TTC GG(A, G) TG(C, G)GTCTTG A |  |  |
| *qnorB* | qnorB-454F | TACTAYGARCCCTGGACTTACRA | 95°C 10 min; 95°C 15 s, 57°C 45 s, 72°C 30 s; 40cycles | (Hirayama et al., 2005) |
|  | qnorB-710R | ATGCGYGGSAWRTAGAAGWAMAMSA |  |  |
| *nosZ* | *nosZ* 1527F | CGCTGTTCHTCGACAGYCA | 95°C 10 min; 95°C 15 s, 58°C 50 s, 72°C 30 s;40cycles | (Scala and Kerkhof, 1998) |

**References**

Bru D, Sarr A, Philippot L. 2007. Relative Abundances of Proteobacterial Membrane-Bound and Periplasmic Nitrate Reductases in Selected Environments. Appl. Environ. Microb. 73, 5971-5974.

Hirayama H, Takai K, Inagaki F, Yamato Y, Suzuki M, Nealson KH, Horikoshi K. 2005. Bacterial community shift along a subsurface geothermal water stream in a Japanese gold mine. Extremophiles. 9, 169-184.

López-Gutiérrez JC, Henry S, Hallet S, Martin-Laurent F, Catroux G, Philippot L. 2004. Quantification of a novel group of nitrate-reducing bacteria in the environment by real-time PCR. J. Microbiol. Meth. 57, 399-407.

Mahnert A, Moissl-Eichinger C, Berg G. 2015. Microbiome interplay: plants alter microbial abundance and diversity within the built environment. Frontiers in microbiology. 6, 887.

Scala DJ, Kerkhof LJ. 1998. Nitrous oxide reductase (nosZ) gene-specific PCR primers for detection of denitrifiers and three nosZ genes from marine sediments. FEMS. microbiol. Lett. 162, 61-68.

Throbäck IN, Enwall K, Jarvis Å, Hallin S. 2004. Reassessing PCR primers targeting nirS, nirK and nosZ genes for community surveys of denitrifying bacteria with DGGE. Fems. Microbiol. Lett. 49, 401-417.

Yan TF, Fields MW, Wu LY, Zu YG, Tiedje JM, Zhou JZ. 2003. Molecular diversity and characterization of nitrite reductase gene fragments (nirK and nirS) from nitrate- and uranium-contaminated groundwater. Environ. Microbiol. 5, 13-24.

**Table S5** Variance analysis data of the abundances of denitrifying functional genes in *Robinia pseudoacacia* plantations sites.

| Genes | *df* | F | P | Adjusted R^2^ |
| --- | --- | --- | --- | --- |
| bacteria | 4 | 21.656 | <0.001 | 0.897 |
| *napA* | 4 | 272.349 | <0.001 | 0.991 |
| *narG* | 4 | 2.673 | 0.095 | 0.517 |
| *nirK* | 4 | 9.314 | 0.002 | 0.788 |
| *nirS* | 4 | 10.135 | 0.002 | 0.802 |
| *qnorB* | 4 | 4.846 | 0.020 | 0.660 |
| *nosZ* | 4 | 10.792 | 0.001 | 0.812 |

Variance analysis was used a general linear model followed by an LSD test through SPSS 20.0. The bacteria, *napA, narG, nirK, nirS, qnorB* and *nosZ* showed the abundances of denitrifying functional genes (copies·g^-1^). *df* = degree of freedom; F = F − value; P = P − value; Adjusted R^2^ = adjusted R-squared value.

**Table S6** Nitrate reducing functional genes carried by selected genera based on Illumina MiSeq high-throughput sequencing database.

| No | Genus | Functional genes |
| --- | --- | --- |
| 1 | *Acanthamoeba* | *qnorB* |
| 2 | *Achromobacter* | *napA, nirS, qnorB, nosZ* |
| 3 | *Acidimicrobium* | *nirS, qnorB* |
| 4 | *Acidithiobacillus* | *qnorB* |
| 5 | *Acidiphilium* | *nirK, nirS* |
| 6 | *Acidobacterium* | *nirK, qnorB* |
| 7 | *Acidothermus* | *nirK* |
| 8 | *Acidovorax* | *nirK, nirS, qnorB, nosZ* |
| 9 | *Acinetobacter* | *bacteria* |
| 10 | *Acrocarpospora* | *bacteria* |
| 11 | *Actinocorallia* | *bacteria* |
| 12 | *Actinomadura* | *bacteria* |
| 13 | *Actinomycetospora* | *bacteria* |
| 14 | *Actinoplanes* | *bacteria, nirK, nirS, qnorB* |
| 15 | *Actinopolymorpha* | *bacteria* |
| 16 | *Actinosynnema* | *nirK, qnorB* |
| 17 | *Adhaeribacter* | *bacteria* |
| 18 | *Aetherobacter* | *bacteria* |
| 19 | *Aeromicrobium* | *bacteria, nirK* |
| 20 | *Agrobacterium* | *bacteria, napA, nirK, nirS, qnorB* |
| 21 | *Agromyces* | *bacteria* |
| 22 | *Alcanivorax* | *nirK, nirS, qnorB* |
| 23 | *Alexandrium* | *nirK, nirS, qnorB* |
| 24 | *Alicyclobacillus* | *bacteria, qnorB* |
| 25 | *Alicycliphilus* | *nirK, nirS, qnorB, nosZ* |
| 26 | *Alistipes* | *nirK* |
| 27 | *Allochromatium* | *nirK, qnorB* |
| 28 | *Alkalipgilus* | *bacteria* |
| 29 | *Amaricoccus* | *bacteria* |
| 30 | *Aminobacter* | *bacteria, qnorB* |
| 31 | *Amycolatopsis* | *bacteria, nirK, nirS, qnorB* |
| 32 | *Anaerobacillus* | *bacteria* |
| 33 | *Anaerolinea* | *bacteria, qnorB* |
| 34 | *Anaeromyxobacter* | *napA, nirK, nirS, qnorB, nosZ* |
| 35 | *Annamia* | *bacteria* |
| 36 | *Ardenscatena* | *bacteria* |
| 37 | *Arenimonas* | *bacteria* |
| 38 | *Aromatoleum* | *nirK, nirS, qnorB, nosZ* |
| 39 | *Arthrobacter* | *nirK, qnorB* |
| 40 | *Arthrospira* | *bacteria* |
| 41 | *Aspergillus* | *qnorB* |
| 42 | *Aquicella* | *bacteria* |
| 43 | *Azoarcus* | *napA, nirK, qnorB, nosZ* |
| 44 | *Azorhizobium* | *nirK, qnorB* |
| 45 | *Azospira* | *nirK, nirS* |
| 46 | *Azospirillum* | *napA, narG, nirK, nirS, qnorB, nosZ* |
| 47 | *Azotobacter* | *bacteria, nirK, qnorB* |
| 48 | *B-42* | *bacteria* |
| 49 | *Bacillus* | *qnorB* |
| 50 | *Balneimonas* | *bacteria* |
| 51 | *Beutenbergia* | *nirK, nirS, qnorB* |
| 52 | *Bdellovibrio* | *bacteria* |
| 53 | *Bifidobacterium* | *qnorB* |
| 54 | *Blastococcus* | *bacteria, nirK, nirS, qnorB* |
| 55 | *Blautia* | *qnorB* |
| 56 | *Bordetella* | *napA, nirK, nirS, qnorB, nosZ* |
| 57 | *Bos* | *qnorB* |
| 58 | *Bosea* | *nirK, nirS* |
| 59 | *Brachybacterium* | *nirK, nirS* |
| 60 | *Bradyrhizobium* | *bacteria, napA, narG, nirK, nirS, qnorB, nosZ* |
| 61 | *Brevibacterium* | *bacteria* |
| 62 | *Brevundimonas* | *nirK, qnorB* |
| 63 | *Brucella* | *nirK, qnorB, nosZ* |
| 64 | *Burkholderia* | *napA, nirK, nirS, qnorB, nosZ* |
| 65 | *Caldilinea* | *nirK, nirS, qnorB, nosZ* |
| 66 | *Candidatus Accumulibacter* | *nirK, qnorB, nosZ* |
| 67 | *Candidatus Entotheonella* | *bacteria* |
| 68 | *Candidatus Koribacter* | *bacteria, nirK, nirS* |
| 69 | *Candidatus Methylomirabilis* | *nirK* |
| 70 | *Candidatus Nitrosospgaera* | *bacteria* |
| 71 | *Candidatus Solibacter* | *bacteria, nirK, nirS, qnorB* |
| 72 | *Candidatus Xiphinrmatobacter* | *bacteria* |
| 73 | *Carnobacterium* | *bacteria* |
| 74 | *Catellatospora* | *bacteria* |
| 75 | *Catenulispora* | *nirK, qnorB* |
| 76 | *Caulobacter* | *bacteria, narG, nirK, nirS, qnorB* |
| 77 | *Cellulomonas* | *bacteria, nirK, qnorB* |
| 78 | *Cellulosimicrobium* | *bacteria* |
| 79 | *Chaetomiun* | *qnorB* |
| 80 | *Chelativorans* | *nirK, nirS, qnorB* |
| 81 | *Chitinophaga* | *bacteria, qnorB* |
| 82 | *Chlamydomonas* | *nirK, qnorB* |
| 83 | *Chloracidobacterium* | *nirK, qnorB* |
| 84 | *Chlorobaculum* | *qnorB* |
| 85 | *Chlorobium* | *qnorB* |
| 86 | *Chloroherpeton* | *qnorB* |
| 87 | *Chloronema* | *bacteria* |
| 88 | *Chlorovirus* | *qnorB* |
| 89 | *Chthoniobacter* | *bacteria* |
| 90 | *Chondromyces* | *bacteria* |
| 91 | *Chromobacterium* | *nirK, qnorB* |
| 92 | *Chromohalobacter* | *qnorB* |
| 93 | *Clavibacter* | *nirK, nirS, qnorB* |
| 94 | *Clostridium* | *bacteria* |
| 95 | *Cohnella* | *bacteria* |
| 96 | *Collimonas* | *qnorB* |
| 97 | *Comamonas* | *napA* |
| 98 | *Conexibacter* | *bacteria, nirK, nirS, qnorB* |
| 99 | *Coprothermobacter* | *bacteria* |
| 100 | *Corallococcus* | *bacteria, nirK, nirS, qnorB* |
| 101 | *Corynebacterium* | *nirK, nirS, qnorB* |
| 102 | *Craurococcus* | *bacteria* |
| 103 | *Crocinitomix* | *bacteria* |
| 104 | *Cronobacter* | *narG* |
| 105 | *Cryptosporangium* | *bacteria* |
| 106 | *Cupriavidus* | *bacteria, nirK, nirS, qnorB, nosZ* |
| 107 | *Curvibacter* | *nirK* |
| 108 | *Cyanobium* | *nirK, qnorB* |
| 109 | *Cylindrospermum* | *qnorB* |
| 110 | *Cystobacter* | *nirK* |
| 111 | *Dactylosporangium* | *bacteria* |
| 112 | *Dechloromonas* | *napA, nosZ* |
| 113 | *Deinococcus* | *nirK, nirS, qnorB* |
| 114 | *Delftia* | *nirK, qnorB* |
| 115 | *Denitratisoma* | *bacteria* |
| 116 | *Desulfarculus* | *nirK, qnorB* |
| 117 | *Desulfobacca* | *qnorB* |
| 118 | *Desulfobulbus* | *nirS* |
| 119 | *Desulfomicrobium* | *qnorB* |
| 120 | *Desulfomonile* | *qnorB* |
| 121 | *Desulfovibrio* | *nirK, nirS* |
| 122 | *Devosia* | *bacteria* |
| 123 | *Diaphorobacter* | *nosZ* |
| 124 | *Dinoroseobacter* | *nirS, qnorB* |
| 125 | *Dyadobacter* | *bacteria, qnorB* |
| 126 | *Ellin506* | *bacteria* |
| 127 | *Elusimicrobium* | *qnorB* |
| 128 | *Eggerthella* | *qnorB* |
| 129 | *Enhydrobacter* | *bacteria* |
| 130 | *Ensifer* | *nirK* |
| 131 | *Enterobacter* | *qnorB* |
| 132 | *Enterococcus* | *bacteria* |
| 133 | *Erwinia* | *nirK* |
| 134 | *Erythrobacter* | *napA, nirK, qnorB* |
| 135 | *Escherichia* | *bacteria, napA, narG, nirK* |
| 136 | *Euzebya* | *bacteria* |
| 137 | *Exiguobacterium* | *qnorB* |
| 138 | *Faecalibacterium* | *nirS, qnorB* |
| 139 | *Ferrimonas* | *napA* |
| 140 | *Fervidobacterium* | *bacteria* |
| 141 | *Fibrella* | *nirK, qnorB* |
| 142 | *Fimbriimonas* | *bacteria* |
| 143 | *Flavisolibacter* | *bacteria* |
| 144 | *Frankia* | *nirK, nirS, qnorB* |
| 145 | *Fretibacterium* | *nirK* |
| 146 | *Fusarium* | *qnorB* |
| 147 | *Gallus* | *qnorB* |
| 148 | *Gemmata* | *bacteria* |
| 149 | *Gemmatimonas* | *bacteria, nirK, nirS, qnorB, nosZ* |
| 150 | *Geobacillus* | *bacteria, nosZ* |
| 151 | *Geobacter* | *nirK, nirS, qnorB* |
| 152 | *Geodermatophilus* | *bacteria, nirK, nirS, qnorB* |
| 153 | *Georgenia* | *bacteria* |
| 154 | *Gloeobacter* | *nirK, qnorB* |
| 155 | *Gluconacetobacter* | *nirK, nirS* |
| 156 | *Gluconobacter* | *qnorB* |
| 157 | *Glycomyces* | *bacteria* |
| 158 | *Gordonia* | *nirK, qnorB* |
| 159 | *Gordonibacter* | *nirK* |
| 160 | *Granulicella* | *nirS, qnorB* |
| 161 | *Hahella* | *qnorB* |
| 162 | *Haliangium* | *bacteria, nirK, nirS, qnorB* |
| 163 | *Haliscomenobacter* | *nosZ* |
| 164 | *Haloarcula* | *nosZ* |
| 165 | *Halobacterium* | *nosZ* |
| 166 | *Haloferax* | *nosZ* |
| 167 | *Halomonas* | *bacteria, qnorB, nosZ* |
| 168 | *Halopiger* | *nirK* |
| 169 | *Halorhodospira* | *nirS, qnorB* |
| 170 | *Haloterrigena* | *nirK* |
| 171 | *Halorthiobacillus* | *qnorB* |
| 172 | *Heliobacterium* | *nirK* |
| 173 | *Herbaspirillum* | *nirK, qnorB, nosZ* |
| 174 | *Herpetosiphon* | *nirK* |
| 175 | *Homo* | *narG, qnorB* |
| 176 | *Hoyosella* | *qnorB* |
| 177 | *Hylemonella* | *bacteria* |
| 178 | *Hymenobacter* | *bacteria* |
| 179 | *Hyphomicrobium* | *bacteria, nirK, nirS, qnorB* |
| 180 | *Hyphomonas* | *nirK, qnorB* |
| 181 | *Ilumatobacter* | *nirK, nirS, qnorB* |
| 182 | *Inquilinus* | *bacteria* |
| 183 | *Intrasporangium* | *nirK, qnorB* |
| 184 | *Isoptericola* | *nirK, qnorB* |
| 185 | *Isosphaera* | *nirK, nirS, qnorB* |
| 186 | *Janthinobacterium* | *bactreia, qnorB* |
| 187 | *JG37-AG-70* | *bacteria* |
| 188 | *Jiangella* | *bacteria* |
| 189 | *Kaistobacter* | *bacteria* |
| 190 | *Kibdelosporangium* | *bacteria* |
| 191 | *Kineococcus* | *nirK, nirS, qnorB* |
| 192 | *Kinetoplastibacterium* | *qnorB* |
| 193 | *Kitasatospora* | *nirK, qnorB* |
| 194 | *Klebsiella* | *bacteria, qnorB* |
| 195 | *Knoellia* | *bacteria* |
| 196 | *Kocuria* | *napA, nirK, nirS* |
| 197 | *Komagataeibacter* | *qnorB* |
| 198 | *Kribbella* | *bacteria, nirK, nirS, qnorB* |
| 199 | *KSA1* | *bacteria* |
| 200 | *Kytococcus* | *nirK, qnorB* |
| 201 | *Labilithrix* | *nosZ* |
| 202 | *Labrys* | *bacteria* |
| 203 | *Lactobacillus* | *bacteria* |
| 204 | *Lactococcus* | *bacteria* |
| 205 | *Lametila* | *nirK* |
| 206 | *Lamia* | *bacteria* |
| 207 | *Laribacter* | *napA, qnorB* |
| 208 | *Legionella* | *bacteria* |
| 209 | *Leisingera* | *nosZ* |
| 210 | *Lentzea* | *bacteria* |
| 211 | *Leptosphaeria* | *qnorB* |
| 212 | *Leptothrix* | *napA, nirK, nirS, qnorB* |
| 213 | *Leuconostoc* | *bacteria* |
| 214 | *Lsoptericola* | *narG* |
| 215 | *Lupinus* | *bacteria* |
| 216 | *Luteimonas* | *bacteria* |
| 217 | *Luteolibacter* | *bacteria* |
| 218 | *Lysobacter* | *bacteria* |
| 219 | *Magnetospirillum* | *nirK, nirS, qnorB, nosZ* |
| 220 | *Marinactinospora* | *qnorB* |
| 221 | *Marinithermus* | *nirK, qnorB* |
| 222 | *Meiothermus* | *nirK, nirS, qnorB* |
| 223 | *Mesorhizobium* | *bacteria, nirK, nirS, qnorB, nosZ* |
| 224 | *Methanoculleus* | *nirK* |
| 225 | *Methanopyrus* | *qnorB* |
| 226 | *Methanoregula* | *nirK* |
| 227 | *Methanosaeta* | *nirK* |
| 228 | *Methanosphaerula* | *qnorB* |
| 229 | *Methylacidiphilum* | *qnorB* |
| 230 | *Methylobacterium* | *bcateria, nirK* |
| 231 | *Methylococcus* | *nirK, nirS* |
| 232 | *Methylibium* | *bcateria, nirK, nirS, qnorB* |
| 233 | *Methylobacterium* | *napA, narG, nirS, qnorB* |
| 234 | *Methylocystis* | *nirK, qnorB* |
| 235 | *Methylopila* | *bacteria* |
| 236 | *Methylobacillus* | *nirK* |
| 237 | *Methylocella* | *nirK, qnorB* |
| 238 | *Methylomicrobium* | *nirS* |
| 239 | *Microbacterium* | *nirK, qnorB* |
| 240 | *Microbispora* | *bacteria* |
| 241 | *Micrococcus* | *nirK* |
| 242 | *Microlunatus* | *narG, nirK, nirS, qnorB* |
| 243 | *Micromonas* | *nirK* |
| 244 | *Micromonospora* | *napA, nirK, nirS, qnorB* |
| 245 | *Missulena* | *qnorB* |
| 246 | *Modestobacter* | *bcateria, narG, nirK, qnorB* |
| 247 | *Mycobacterium* | *bacteria, nirK, nirS, qnorB* |
| 248 | *Mycoplana* | *bacteria* |
| 249 | *Myxococcus* | *bacteria, nirK, nirS, qnorB, nosZ* |
| 250 | *Nakamurella* | *nirK, qnorB* |
| 251 | *Nannocystis* | *bacteria* |
| 252 | *Natrinema* | *nirK* |
| 253 | *Natronococcus* | *nirK* |
| 254 | *Natronomonas* | *nirK* |
| 255 | *Niastella* | *bacteria, qnorB* |
| 256 | *Nitrobacter* | *nirK, nirS, qnorB* |
| 257 | *Nitrosococcus* | *qnorB* |
| 258 | *Nitrosomonas* | *qnorB* |
| 259 | *Nitrosospaera* | *qnorB* |
| 260 | *Nitrosospira* | *nirK* |
| 261 | *Nitrospira* | *bacteria, nirK* |
| 262 | *Nocardiopsis* | *nirK, qnorB* |
| 263 | *Nocardia* | *bacteria, narG, nirK, qnorB* |
| 264 | *Nocardioides* | *bacteria, nirK, nirS, qnorB* |
| 265 | *Nonomuraea* | *bacteria, nirK* |
| 266 | *Nostoc* | *qnorB* |
| 267 | *Novosphingobium* | *bacteria, nirK, nirS, qnorB* |
| 268 | *Oceanimonas* | *napA* |
| 269 | *Oceanithermus* | *nirK, qnorB* |
| 270 | *Ochrobactrum* | *bacteria, nosZ* |
| 271 | *Oligotropha* | *nirK, qnorB, nosZ* |
| 272 | *Olsenella* | *nirS* |
| 273 | *Opitutus* | *bacteria, nirK, nirS, qnorB, nosZ* |
| 274 | *OR-59* | *bacteria* |
| 275 | *Oscillibacter* | *qnorB* |
| 276 | *Oscillochloris* | *bacteria* |
| 277 | *Paenibacillus* | *bacteria* |
| 278 | *Paeniporosarcina* | *bacteria* |
| 279 | *Pantoea* | *narG, qnorB* |
| 280 | *Paraburkholderia* | *nirK* |
| 281 | *Paracoccus* | *napA, nirK, qnorB, nosZ* |
| 282 | *Parvibaculum* | *nirS* |
| 283 | *Pedobacter* | *bacteria* |
| 284 | *Pedomicrobium* | *bacteria* |
| 285 | *Pedosphaera* | *bacteria* |
| 286 | *Pelagibacterium* | *nirK, nirS, qnorB* |
| 287 | *Persephonella* | *qnorB* |
| 288 | *Phenylobacterium* | *bacteria, nirK, nirS, qnorB* |
| 289 | *Phormidium* | *bacteria* |
| 290 | *Phycicoccus* | *bacteria* |
| 291 | *Phycisphaera* | *nirK, nirS, qnorB* |
| 292 | *Phyllobacterium* | *bacteria* |
| 293 | *Phytohabitans* | *bacteria* |
| 294 | *Pilimelia* | *bacteria* |
| 295 | *Pimelobacter* | *bacteria* |
| 296 | *Pirellula* | *bacteria, nirK, nirS, qnorB* |
| 297 | *Planctopirus* | *nirK, qnorB* |
| 298 | *Planctomyces* | *bacteria* |
| 299 | *Pleomorphomonas* | *bacteria* |
| 300 | *Plesiocystis* | *bacteria* |
| 301 | *Polaromonas* | *bacteria, nirK, qnorB* |
| 302 | *Polymorphum* | *nirK, nirS, qnorB, nosZ* |
| 303 | *Pontibacter* | *bacteria* |
| 304 | *Porphyromonas* | *qnorB* |
| 305 | *Prevotella* | *bacteria* |
| 306 | *Promicromonospora* | *bacteria* |
| 307 | *Promurmekiaphila* | *qnorB* |
| 308 | *Propionibacterium* | *nosZ* |
| 309 | *Prosthecobacter* | *bacteria* |
| 310 | *Providencia* | *napA* |
| 311 | *Pseudarthrobacter* | *nirK* |
| 312 | *Pseudogulbenkiania* | *nirK, nosZ* |
| 313 | *Pseudomonas* | *bacteria, napA, narG, nirK, nirS, qnorB, nosZ* |
| 314 | *Pseudonocardia* | *bacteria, nirK, nirS, qnorB* |
| 315 | *Pseudoxanthomonas* | *napA, narG, nirK, nirS, qnorB* |
| 316 | *Psychromonas* | *nosZ* |
| 317 | *Pterygota* | *napA, nirK, qnorB* |
| 318 | *Pusillimonas* | *napA* |
| 319 | *Rahnella* | *narG, nirK* |
| 320 | *Ralstonia* | *bacteria, nirK, nirS, qnorB, nosZ* |
| 321 | *Ramlibacter* | *bacteria, narG, nirK, nirS, qnorB* |
| 322 | *Renibacterium* | *nirK* |
| 323 | *Rhizobium* | *napA, nirK, nirS, qnorB, nosZ* |
| 324 | *Rhodobacter* | *napA, nirK, nirS, qnorB, nosZ* |
| 325 | *Rhodanobacter* | *narG, nirK, qnorB, nosZ* |
| 326 | *Rhodococcus* | *bacteria, nirK, nirS, qnorB* |
| 327 | *Rhodocytophaga* | *bacteria* |
| 328 | *Rhodoferax* | *nirK* |
| 329 | *Rhodomicrobium* | *nirK, nirS, qnorB* |
| 330 | *Rhodopirellila* | *qnorB* |
| 331 | *Rhodoplanes* | *bacteria* |
| 332 | *Rhodopseudomonas* | *napA, narG, nirK, nirS, qnorB, nosZ* |
| 333 | *Rhodospirillum* | *napA, nirK, nirS, qnorB, nosZ* |
| 334 | *Rhodothermus* | *nirK, nosZ* |
| 335 | *Ricinus* | *qnorB* |
| 336 | *Robiginitalea* | *qnorB, nosZ* |
| 337 | *Roseiflexus* | *bacteria, nirK, qnorB* |
| 338 | *Roseobacter* | *nirS, qnorB* |
| 339 | *Roseburia* | *qnorB* |
| 340 | *Roseomonas* | *bacteria* |
| 341 | *Rubellimicrobium* | *bacteria* |
| 342 | *Rubinisphaera* | *nirK, qnorB* |
| 343 | *Rubricoccus* | *bacteria* |
| 344 | *Rubrivivax* | *bacteria, nirK, nirS, qnorB, nosZ* |
| 345 | *Rubrobacter* | *bacteria, nirK, nirS, qnorB* |
| 346 | *Runella* | *qnorB* |
| 347 | *Saccharomonospora* | *nirK* |
| 348 | *Saccharopolyspora* | *bacteria, nirK, qnorB* |
| 349 | *Saccharothrix* | *bacteria, narG, nirK, nirS, qnorB* |
| 350 | *Salinarcheaum* | *qnorB* |
| 351 | *Salinibacterium* | *bacteria* |
| 352 | *Salinispora* | *nirK, qnorB* |
| 353 | *Salmonella* | *napA, narG, qnorB* |
| 354 | *Salpingoeca* | *qnorB* |
| 355 | *Sanguibacter* | *nirK, qnorB* |
| 356 | *Schizophyllum* | *qnorB* |
| 357 | *Scincella* | *qnorB* |
| 358 | *Sediminibacterium* | *bacteria* |
| 359 | *Segetibacter* | *bacteria* |
| 360 | *Selenomonas* | *nirK, nirS* |
| 361 | *Serratia* | *napA, narG, nirK, qnorB* |
| 362 | *Shewanella* | *napA, qnorB* |
| 363 | *Shigella* | *napA, nosZ* |
| 364 | *Sideroxydans* | *nirK* |
| 365 | *Singulisphaera* | *napA, narG, nirK, nirS, qnorB* |
| 366 | *Sinorhizobium* | *bacteria, napA, nirK, nirS, qnorB, nosZ* |
| 367 | *Skermanella* | *bacteria* |
| 368 | *Slackia* | *qnorB* |
| 369 | *Solibacillus* | *bacteria* |
| 370 | *Solirubrobacter* | *bacteria* |
| 371 | *Solitalea* | *qnorB* |
| 372 | *Solwaraspora* | *bacteria* |
| 373 | *Sorangium* | *bacteria, nirK, nirS, qnorB* |
| 374 | *Sphaerisporangium* | *bacteria* |
| 375 | *Sphaerobacter* | *nirK, nirS, qnorB, nosZ* |
| 376 | *Sphingobium* | *nirK, nirS, qnorB* |
| 377 | *Sphingomonas* | *bacteria, narG, nirK, nirS, qnorB* |
| 378 | *Sphingopyxis* | *bacteria, nirK, nirS, qnorB* |
| 379 | *Spiribacter* | *qnorB* |
| 380 | *Spirillospora* | *bacteria* |
| 381 | *Spirochaeta* | *nirK, qnorB* |
| 382 | *Sporichthya* | *bacteria* |
| 383 | *Sporocytophaga* | *bacteria* |
| 384 | *Stackebrandtia* | *nirK, nirS, qnorB* |
| 385 | *Starkeya* | *napA, nirK, nirS, qnorB* |
| 386 | *Stenotrophomonas* | *napA, nirK* |
| 387 | *Steroidobacter* | *bacteria* |
| 388 | *Stigmatella* | *nirK, nirS, qnorB* |
| 389 | *Streptacidiphilus* | *bacteria, nirS, qnorB* |
| 390 | *Streptococcus* | *bacteria* |
| 391 | *Streptomyces* | *bacteria, narG, nirK, nirS, qnorB, nosZ* |
| 392 | *Streptosporangium* | *nirK, nirS, qnorB* |
| 393 | *Sulfuricella* | *nosZ* |
| 394 | *Sulfuritalea* | *nosZ* |
| 395 | *Sulfurospirillum* | *qnorB* |
| 396 | *Symbiobacterium* | *narG, nirK, nirS* |
| 397 | *Synechococcus* | *qnorB* |
| 398 | *Syntrophobacter* | *nirK* |
| 399 | *Terracoccus* | *bacteria* |
| 400 | *Terriglobus* | *nirS, qnorB* |
| 401 | *Thalassobaculum* | *nosZ* |
| 402 | *Thauera* | *napA, nirK, nirS, qnorB, nosZ* |
| 403 | *Thermacetogenium* | *qnorB* |
| 404 | *Thermaerobacter* | *nirK, qnorB* |
| 405 | *Thermobacillus* | *nirK, qnorB* |
| 406 | *Thermobaculum* | *bacteria, nirK* |
| 407 | *Thermobispora* | *nirK, qnorB* |
| 408 | *Thermobifida* | *nirK* |
| 409 | *Thermomicrobium* | *nirK, nosZ* |
| 410 | *Thermomonas* | *bacteria* |
| 411 | *Thermomonospra* | *nirK, qnorB* |
| 412 | *Thermovibrio* | *qnorB* |
| 413 | *Thermus* | *nirK, nirS, qnorB* |
| 414 | *Thioalkalivibrio* | *nirK, nirS, qnorB* |
| 415 | *Thiobacillus* | *nirK, nirS, qnorB* |
| 416 | *Thiocystis* | *nirS* |
| 417 | *Thioflavicoccus* | *nirK, qnorB* |
| 418 | *Thiomonas* | *nirK, qnorB* |
| 419 | *Tistrella* | *nirK, qnorB* |
| 420 | *Treponema* | *qnorB* |
| 421 | *Triticum* | *nirK* |
| 422 | *Truepera* | *bacteria, nirK, qnorB* |
| 423 | *Tsukamurella* | *qnorB* |
| 424 | *Variovorax* | *bacteria, nirK, nirS, qnorB* |
| 425 | *Vermamoeba* | *bacteria* |
| 426 | *Verminephrobacter* | *napA, nirS* |
| 427 | *Verrucosispora* | *nirK, nirS, qnorB* |
| 428 | *Vertebrata* | *nirK* |
| 429 | *Virgisporangium* | *bacteria* |
| 430 | *Xylanimicrobium* | *bacteria* |
| 431 | *Xylanimonas* | *nirK, qnorB* |
| 432 | *Xanthobacter* | *nirK, nirS, qnorB* |
| 433 | *Xanthomonas* | *nirK, nirS, qnorB* |

**Table S7** The features of denitrifier communities in *Robinia pseudoacacia* plantations sites.

| Index | sites | bacteria | *napA* | *narG* | *nirK* | *nirS* | *qnorB* | *nosZ* |
| --- | --- | --- | --- | --- | --- | --- | --- | --- |
| Chao 1 | 0-y | 2405 | 2294 | 240 | 2398 | 667 | 1185 | 625 |
|  | 14-y | 2644 | 2427 | 225 | 2175 | 558 | 1393 | 530 |
|  | 26-y | 3168 | 2995 | 249 | 2710 | 705 | 1652 | 585 |
|  | 33-y | 2752 | 2346 | 248 | 3105 | 508 | 1408 | 505 |
|  | 42-y | 2850 | 2546 | 275 | 2829 | 621 | 3017 | 667 |
| Simpson index | 0-y | 1.00 | 0.98 | 0.78 | 0.97 | 0.97 | 0.97 | 0.97 |
|  | 14-y | 1.00 | 0.98 | 0.74 | 0.94 | 0.97 | 0.99 | 0.96 |
|  | 26-y | 1.00 | 0.98 | 0.83 | 0.99 | 0.98 | 1.00 | 0.97 |
|  | 33-y | 1.00 | 0.94 | 0.72 | 0.97 | 0.97 | 0.99 | 0.93 |
|  | 42-y | 1.00 | 0.96 | 0.92 | 1.00 | 0.97 | 1.00 | 0.97 |
| Shannon index | 0-y | 0.99 | 0.89 | 0.55 | 0.80 | 0.92 | 0.90 | 0.81 |
|  | 14-y | 1.00 | 0.91 | 0.51 | 0.79 | 0.88 | 0.94 | 0.78 |
|  | 26-y | 1.01 | 0.93 | 0.59 | 0.82 | 0.99 | 0.96 | 0.81 |
|  | 33-y | 0.99 | 0.85 | 0.53 | 0.79 | 0.93 | 0.94 | 0.75 |
|  | 42-y | 0.99 | 0.88 | 0.69 | 0.80 | 0.99 | 1.01 | 0.81 |

Chao 1 index show the richness, Simpson index show the evenness and Shannon index show the diversity of the denitrifier communities.

**Table S8** The information of bacterial and denitrifiers communities through Illumina MiSeq high-throughput sequencing in *Robinia pseudoacacia* plantations sites.

| Phylum | bacteria | *napA* | *narG* | *nirK* | *nirS* | *qnorB* | *nosZ* |
| --- | --- | --- | --- | --- | --- | --- | --- |
| *Actinobacteria* | 23.15% | − | − | 16.84% | − | 8.88% | − |
| *Proteobacteria* | 21.67% | 41.77% | 55.98% | 15.70% | 5.81% | 12.84% | 89.80% |
| *Acidobacteria* | 17.16% | − | − | − | − | − | − |
| *Planctomycetes* | 12.79% | − | − | − | − | − | − |
| *Gemmatimonadetes* | 7.18% | − | − | − | − | − | − |
| *Chloroflexi* | 7.15% | − | − | − | − | − | − |

The six phyla above were the major bacteria communities and the two phyla were the major denitrifiers communities. The percentage show the average of all sites.

**Table S9** The information of bacterial communities through Illumina MiSeq high-throughput sequencing in *Robinia pseudoacacia* plantations sites.

| Phylum | 0-y | 14-y | 26-y | 33-y | 42-y |
| --- | --- | --- | --- | --- | --- |
| *Actinobacteria* | 33.75% | 14.55% | 20.82% | 27.28% | 19.37% |
| *Proteobacteria* | 17.83% | 33.53% | 21.35% | 21.21% | 14.43% |
| *Acidobacteria* | 10.47% | 16.85% | 19.65% | 20.14% | 18.68% |
| *Planctomycetes* | 7.81% | 10.91% | 15.64% | 7.25% | 22.33% |
| *Gemmatimonadetes* | 5.17% | 7.96% | 7.77% | 7.27% | 7.73% |
| *Chloroflexi* | 9.81% | 7.83% | 5.39% | 7.37% | 5.36% |

The percentage show the each sites in the six major phyla of bacterial communities.

**Table S10** Multiple regression analysis for the gene fragments on N_2_O emissiom rates among artificial restoration stages. The adjusted R^2^ was 0.53 at a significance of 0.018.

$$N_{2}O=38.781+3.431\times\frac{qnorB}{nirK}-2.668\times\frac{nosZ}{nirS}-17.136\times\frac{napA+narG}{Bacteria}-5.05\times\frac{nirK+nirS}{napA+narG}$$

The bacteria, *napA, narG, nirK, nirS, qnorB* and *nosZ* showed the abundances of denitrifying functional genes (copies·g^-1^).

**Table S11** Correlation analysis among soil properties, N_2_O emission rates and denitrifying bacterial community in *Robinia pseudoacacia* plantations sites.

|  | N_2_O | napA-1 | napA-2 | napA-3 | narG-1 | narG-2 | narG-3 | nirK-1 | nirK-2 | nirK-3 | nirS-1 | nirS-2 | nirS-3 | qnorB-1 | qnorB-2 | qnorB-3 | nosZ-1 | nosZ-2 | nosZ-3 | BD | pH | OC | TN | ammonium |
| --- | --- | --- | --- | --- | --- | --- | --- | --- | --- | --- | --- | --- | --- | --- | --- | --- | --- | --- | --- | --- | --- | --- | --- | --- |
| napA-1 | .616^*^ |  |  |  |  |  |  |  |  |  |  |  |  |  |  |  |  |  |  |  |  |  |  |  |
| napA-2 | .488 | .978^**^ |  |  |  |  |  |  |  |  |  |  |  |  |  |  |  |  |  |  |  |  |  |  |
| napA-3 | .679^**^ | -.151 | -.283 |  |  |  |  |  |  |  |  |  |  |  |  |  |  |  |  |  |  |  |  |  |
| narG-1 | .720^**^ | .304 | .224 | .697^**^ |  |  |  |  |  |  |  |  |  |  |  |  |  |  |  |  |  |  |  |  |
| narG-2 | .609^*^ | .092 | .042 | .758^**^ | .958^**^ |  |  |  |  |  |  |  |  |  |  |  |  |  |  |  |  |  |  |  |
| narG-3 | -.034 | -.289 | -.435 | .222 | .154 | .042 |  |  |  |  |  |  |  |  |  |  |  |  |  |  |  |  |  |  |
| nirK-1 | .439 | .007 | -.011 | .640^*^ | .914^**^ | .977^**^ | .034 |  |  |  |  |  |  |  |  |  |  |  |  |  |  |  |  |  |
| nirK-2 | -.946^**^ | -.570^*^ | -.461 | -.629^*^ | -.504 | -.432 | .230 | -.242 |  |  |  |  |  |  |  |  |  |  |  |  |  |  |  |  |
| nirK-3 | -.594^*^ | -.917^**^ | -.942^**^ | .063 | -.494 | -.358 | .465 | -.317 | .520^*^ |  |  |  |  |  |  |  |  |  |  |  |  |  |  |  |
| nirS-1 | .726^**^ | .291 | .244 | .714^**^ | .964^**^ | .975^**^ | -.090 | .929^**^ | -.572^*^ | -.537^*^ |  |  |  |  |  |  |  |  |  |  |  |  |  |  |
| nirS-2 | .406 | .256 | .095 | .175 | -.190 | -.353 | .377 | -.527^*^ | -.485 | .115 | -.287 |  |  |  |  |  |  |  |  |  |  |  |  |  |
| nirS-3 | -.658^**^ | -.845^**^ | -.733^**^ | -.042 | -.434 | -.163 | -.262 | -.061 | .506 | .668^**^ | -.291 | -.489 |  |  |  |  |  |  |  |  |  |  |  |  |
| qnorB-1 | .035 | .446 | .369 | -.369 | .035 | -.226 | .605^*^ | -.222 | .160 | -.214 | -.200 | .368 | -.749^**^ |  |  |  |  |  |  |  |  |  |  |  |
| qnorB-2 | -.422 | .408 | .507 | -.939^**^ | -.668^**^ | -.784^**^ | -.300 | -.731^**^ | .336 | -.243 | -.675^**^ | .079 | -.184 | .421 |  |  |  |  |  |  |  |  |  |  |
| qnorB-3 | -.597^*^ | -.618^*^ | -.633^*^ | -.143 | -.088 | -.077 | .757^**^ | .052 | .779^**^ | .621^*^ | -.269 | -.215 | .235 | .372 | -.130 |  |  |  |  |  |  |  |  |  |
| nosZ-1 | .389 | .957^**^ | .992^**^ | -.394 | .109 | -.073 | -.452 | -.114 | -.378 | -.903^**^ | .130 | .095 | -.695^**^ | .390 | .608^*^ | -.608^*^ |  |  |  |  |  |  |  |  |
| nosZ-2 | -.320 | -.932^**^ | -.978^**^ | .441 | -.152 | .034 | .392 | .046 | .267 | .906^**^ | -.150 | .010 | .697^**^ | -.459 | -.613^*^ | .486 | -.986^**^ |  |  |  |  |  |  |  |
| nosZ-3 | .050 | .783^**^ | .876^**^ | -.669^**^ | -.232 | -.363 | -.585^*^ | -.359 | -.112 | -.726^**^ | -.176 | -.011 | -.428 | .286 | .828^**^ | -.545^*^ | .926^**^ | -.912^**^ |  |  |  |  |  |  |
| BD | .632^*^ | .509 | .364 | .350 | .692^**^ | .476 | .574^*^ | .403 | -.383 | -.419 | .504 | .327 | -.846^**^ | .683^**^ | -.242 | .117 | .293 | -.337 | -.039 |  |  |  |  |  |
| pH | -.642^**^ | -.922^**^ | -.905^**^ | .026 | -.511 | -.328 | .239 | -.270 | .524^*^ | .933^**^ | -.494 | -.054 | .799^**^ | -.398 | -.221 | .503 | -.863^**^ | .870^**^ | -.647^**^ | -.567^*^ |  |  |  |  |
| OC | -.989^**^ | -.580^*^ | -.431 | -.691^**^ | -.679^**^ | -.552^*^ | -.076 | -.373 | .935^**^ | .511 | -.659^**^ | -.512 | .682^**^ | -.099 | .428 | .537^*^ | -.334 | .262 | .008 | -.668^**^ | .581^*^ |  |  |  |
| TN | -.336 | .523^*^ | .642^**^ | -.912^**^ | -.490 | -.604^*^ | -.411 | -.537^*^ | .286 | -.440 | -.479 | -.094 | -.240 | .394 | .965^**^ | -.199 | .729^**^ | -.751^**^ | .911^**^ | -.180 | -.377 | .371 |  |  |
| ammonium | .719^**^ | .297 | .227 | .703^**^ | .997^**^ | .970^**^ | .093 | .928^**^ | -.516^*^ | -.505 | .979^**^ | -.225 | -.394 | -.026 | -.674^**^ | -.129 | .111 | -.150 | -.222 | .642^**^ | -.508 | -.671^**^ | -.490 |  |
| nitrate | .482 | .858^**^ | .783^**^ | -.191 | .286 | .011 | .199 | -.054 | -.333 | -.680^**^ | .144 | .395 | -.961^**^ | .812^**^ | .387 | -.183 | .770^**^ | -.789^**^ | .571^*^ | .743^**^ | -.800^**^ | -.496 | .445 | .250 |
